# Transcriptome and genome evolution during HER2-amplified breast neoplasia

**DOI:** 10.1186/s13058-021-01451-6

**Published:** 2021-07-15

**Authors:** Peipei Lu, Joseph Foley, Chunfang Zhu, Katherine McNamara, Korsuk Sirinukunwattana, Sujay Vennam, Sushama Varma, Hamid Fehri, Arunima Srivastava, Shirley Zhu, Jens Rittscher, Parag Mallick, Christina Curtis, Robert West

**Affiliations:** 1 Department of Pathology, Stanford University, Stanford, CA USA; 2 Department of Medicine and Genetics, Stanford University, Stanford, CA USA; 3 Institute of Biomedical Engineering, Department of Engineering Science, University of Oxford, Oxford, UK; 4 Big Data Institute/Li Ka Shing Centre for Health Information and Discovery, University of Oxford, Oxford, UK; 5 Department of Computer Science and Engineering, The Ohio State University, Columbus, OH USA; 6 Department of Radiology, Stanford University, Stanford, CA USA

**Keywords:** Oncogene Genomic evolution, ERBB2/HER2, Breast neoplasia

## Abstract

**Background:**

The acquisition of oncogenic drivers is a critical feature of cancer progression. For some carcinomas, it is clear that certain genetic drivers occur early in neoplasia and others late. Why these drivers are selected and how these changes alter the neoplasia’s fitness is less understood.

**Methods:**

Here we use spatially oriented genomic approaches to identify transcriptomic and genetic changes at the single-duct level within precursor neoplasia associated with invasive breast cancer. We study HER2 amplification in ductal carcinoma in situ (DCIS) as an event that can be both quantified and spatially located via fluorescence in situ hybridization (FISH) and immunohistochemistry on fixed paraffin-embedded tissue.

**Results:**

By combining the HER2-FISH with the laser capture microdissection (LCM) Smart-3SEQ method, we found that HER2 amplification in DCIS alters the transcriptomic profiles and increases diversity of copy number variations (CNVs). Particularly, interferon signaling pathway is activated by HER2 amplification in DCIS, which may provide a prolonged interferon signaling activation in HER2-positive breast cancer. Multiple subclones of HER2-amplified DCIS with distinct CNV profiles are observed, suggesting that multiple events occurred for the acquisition of HER2 amplification. Notably, DCIS acquires key transcriptomic changes and CNV events prior to HER2 amplification, suggesting that pre-amplified DCIS may create a cellular state primed to gain HER2 amplification for growth advantage.

**Conclusion:**

By using genomic methods that are spatially oriented, this study identifies several features that appear to generate insights into neoplastic progression in precancer lesions at a single-duct level.

**Supplementary Information:**

The online version contains supplementary material available at 10.1186/s13058-021-01451-6.

## Background

Genomic studies have identified common driver genes and mutations mostly in invasive carcinoma [1–4]. However, many mutations are known to occur at the precancer stage [5, 6], and the biologic benefits that accompany these events are not well understood in the context of the precancer environment, which is significantly different from that of invasive carcinoma. One challenge in the study of carcinogenesis is to capture the timing and location of the mutations that are required for tumor progression to occur. The precancer lesions are particularly difficult to study due to their small size, which requires microscopic identification usually on fixed tissue, resulting in a low-input and low-quality material for genomic methods.

Observing the changes that occur in these precursor lesions will give us better insights into the biology of carcinogenesis. In this study, we use the amplification of ERBB2/HER2 oncogene in breast precancer as a model system, not only because it is known to drive HER2-amplified breast cancer development and progression, but also it is acquired at the pre-invasive stages such as atypical ductal hyperplasia (ADH) and ductal carcinoma in situ (DCIS) [7]. In addition, HER2 gene is amplified in a dose-dependent manner and we can microscopically quantify its copy number using fluorescence in situ hybridization (FISH), rendering it a good model to explore how oncogene changes affect neoplasia progression.

The breast ductal system is contiguous, with ducts branching from the nipple and terminating in lobules where milk is produced [8]. The individual in situ ducts within a large DCIS lesion are derived from one neoplastic process and are clonally related. Sub-sampling of invasive breast carcinoma has been performed in prior publications and has been demonstrated to provide insight into the evolution of breast cancer at the invasive stage [9, 10]. In this study, we sub-sampled DCIS ducts at different regions within a large lesion, aiming to identify the changes by HER2 amplification and uncover the evolutionary path during HER2 amplification in neoplasia progression. However, these in situ ducts, which can only be identified in clinical archival tissues and are difficult to categorize by morphology, are challenging to capture by the conventional single-cell RNA sequencing techniques. To overcome these technical challenges, we recently developed a new kind of RNA sequencing method for archival samples called Smart-3SEQ, which allows us to study genetic profiles of individual cells/ducts with their morphology and spatial context preserved [11]. The Smart-3SEQ data can be combined with other in situ profiles such as FISH and immunohistochemistry on the same ducts using adjacent tissue sections. This new approach enables us to profile multiple microscopic ducts that have different HER2 status within a single lesion, thus allowing us to identify the changes by HER2 amplification and uncover the evolutionary path during HER2 amplification in neoplasia progression.

Our study found that HER2 amplification altered the transcriptomic profiles in DCIS, and particularly, interferon signaling pathway was activated by HER2 amplification in DCIS, which may provide a prolonged interferon signaling activation in HER2-positive breast cancer. The clonal relationship constructed by their CNV profiles showed that DCIS ducts with HER2 amplification were evolved from their surrounding DCIS ducts that lack HER2 amplification. In addition, this evolutionary progression involved multiple subclones of HER2-amplified DCIS with distinct CNV profiles, suggesting that multiple events occurred for the acquisition of HER2 amplification. Notably, pre-existing transcriptomic and genomic changes were observed in pre-amplified DCIS, indicating that pre-amplified DCIS may create a cellular state primed to gain HER2 amplification for growth advantage. We believe this is the first study ever to examine the transcriptomic and genomic profiles during oncogene amplification in precancer lesions at a single duct level.

## Methods

### Samples

DCIS and normal breast samples were collected using HIPAA compliant Stanford University Medical Center institutional review board approved written informed consent. The FFPE tissue blocks were archived with the Stanford University and Oregon Health and Sciences University Departments of Pathology. Histopathological sections of breast resection specimens were screened for the presence of large amounts of DCIS by RBW.

### Immunohistochemistry

IHC was performed on 4-μm FFPE sections using the Ventana BenchMark XT automated immunostaining platform (Ventana Medical Systems/Roche, Tucson, AZ, USA). 25 HER2-positive patients processing large amounts of DCIS were selected for HER2-IHC. Primary antibody was directed toward HER2 (Ventana, Tucson, AZ, USA, catalog #790-2991). Sections of 4-μm were cut from the tissue array blocks, deparaffinized in xylene, hydrated in a graded series of alcohol, and prepared for staining using Target Retrieval Solution, Citrate pH6 (Dako/Agilent, Carpinteria, CA, USA, catalog #S2369) to retrieve antigenic sites at 116°C for 3 minutes. Staining was then performed using the EnVision+ anti-rabbit system (Dako/Agilent, catalog #K4011). Cells staining positive within the nucleus of luminal cells in the epithelial compartment were considered HER2-positive.

### Fluorescence in situ hybridization

FISH was performed to examine HER2 amplification using 4-μm FFPE sections from 6 of the HER2-positive patients that showed heterogeneity of HER2-IHC in DCIS. BAC clones RP11-94L15 (17q12) were obtained from the BACPAC Resources Centre (Children’s Hospital Oakland Research Institute, Oakland, CA), while clone CTD-2344F21 (2q37) was from Invitrogen/Life Technologies (Grand Island, NY, USA). Probe RP11-94L15 was labeled with Cy3 dUTP and control probe CTD-2344F21 were labeled with Cy5 dUTP (Amersham/GE Healthcare, Pittsburgh, PA, USA), using Nick Translation Kit (Abbott Molecular). Slides were deparaffinized in xylene×3 for 10 min, dehydrated twice with 100% ethanol air-dried for 10 min, and then pretreated in a 10mM citric acid pH6 at 80°C for 45 min. Slides were digested for 75min in pepsin (at 75,000 units, Sigma cat# P6887) at 37°C. Fluorescent labeled probes and slides were co-denatured at 75°C for 7min and hybridized at 37°C for 16–18 h in a humidified chamber. Post-hybridization washes were performed using 2×SSC/0.3% NP-40 at 72°C for 5min. Slides were dehydrated and air-dried in dark and counterstained with DAPI (cat# P36935 Invitrogen/Life technologies). Imaging and analysis were performed using Ariol 3.4v software (Leica Microsystems). Fluorescence was recorded using filters 550 (green: 550nm Cy3) and 647 (red: 647nm Cy5).

### HER2-FISH analysis

An automated pipeline is a composite of several computational modules (Supplementary Figure 1) [12]. DCIS boundaries for LCM dissection are first drawn (DCIS segmentation). Individual nuclei are then identified regardless of their cell types (nuclear segmentation). Due to the pepsin treatment, nuclei on FISH images are enlarged, often making individual nuclei indistinguishable if clumped together. An extra computational step is required to better identify individual nuclei (nuclear detection). Next, nuclei are categorized and only those belonging to the epithelial class are considered (nuclear classification). Fluorescent signals inside the epithelial nuclear boundaries are identified (probe detection) and counted (signal counting). The outputs of the pipeline are counts of Cy3- and Cy5-labeled signals per cell, enabling identification of copy number alterations at a single-cell level. All detected epithelial cells within the DCIS boundaries are counted, and signal distribution are recorded.

We counted the absolute number of signals in every detected epithelial cell and recorded them as signal/cell. We used Bayesian model to determine if a cell with certain signals is HER2-amplified or not. An uninformative prior probability (i.e., both events are equally likely) is used to model if a chosen cell is non-amplified or amplified. Probability density of amplified/non-amplified signals were determined by hand-picking the reference samples (18 non-amplified DCIS and 29 amplified DCIS) and combining their signal counts into one reference for each group. The reference samples were selected from all samples involved in this study, and only those with clear non-amplified/amplified features on HER2-FISH image were selected. The probability of the signals was calculated at 0–10 signal/cell for non-amplified/amplified references (Supplementary Figure 2). For a given cell with a certain signal, the posterior probability for that cell being non-amplified/amplified was calculated using the Bayesian formula, and the classification was assigned by the highest probability to which class the cell belongs to (Supplementary Table 1). Due to the high cellular heterogeneity of HER2-FISH signal in DCIS cells (Supplementary Figure 3), the grouping of DCIS ducts was determined by their distribution of HER2-FISH signals at 0-10 signal/cell in all cells for each duct. The distribution of the signals for a given sample was estimated by kernel density estimation (KDE), which used a non-parametric way to estimate the probability density function of a random signal. PCA on the probability density was performed to identify the separation among DCIS groups (Supplementary Figure 4). Samples that are ambiguous between DCIS_noamp and DCIS_amp were defined as DCIS_int (Supplementary Figure 4).

### Laser-capture microdissection

Consecutive sections of the FFPE block were cut on a microtome at 7-μm thickness and mounted on glass slides with polyethylene naphthalate membranes (Thermo Fisher Scientific LCM0522). Slides were immersed 20 s each in xylenes×3, 100% ethanol×3, 95% ethanol×2, 70% ethanol×2, water, hematoxylin (Dako S3309), water, bluing reagent (Thermo Fisher Scientific 7301), water, 70% ethanol×2, 95% ethanol×2, and 100% ethanol×3, xylenes×3. Slides were dissected immediately after staining. Cells were dissected on an ArcturusXT LCM System using both the ultraviolet (UV) laser to cut out each sample and the infrared laser to adhere it to a CapSure HS LCM Cap (Thermo Fisher Scientific LCM0215). Roughly 500 cells were captured by area, according to density estimates by cell counting on small areas. After LCM, the cap was sealed in a 0.5-mL tube (Thermo Fisher Scientific N8010611) and subjected to Smart-3SEQ or DNA library preparation.

### Smart-3SEQ library preparation and sequencing

Smart-3SEQ sequencing libraries from FFPE cells were prepared and pooled according to version 1.9 of the Smart-3SEQ protocol [11]. Briefly, the pre-SPRI pooling option was used for a single batch for all LCM samples, and 22 PCR cycles were used for library amplification. Libraries were profiled for size distribution on an Agilent 2200 TapeStation with High Sensitivity D1000 reagent kits and quantified by qPCR with a dual-labeled probe [13], and libraries were mixed to equimolarity according to the qPCR measurements. Libraries were sequenced with Illumina NextSeq 500 instrument with a High Output v2 reagent kit (Illumina FC-404-2005). The 76-base directional, single-end sequencing reads were obtained and uniquely mapped to hg38 using STAR with the settings provided in the previous protocol [11].

### DNA library preparation and light-pass WGS

We chose DCIS/IDC samples with distinct degrees of HER2 amplification from 4 patients for WGS analysis. LCM slides consecutive to the Smart-3SEQ LCM slides were cut and matched samples were collected to a CapSure HS LCM Cap. Genomic DNA was isolated from FFPE cells using PicoPure DNA Extraction kit (Thermo Fisher Scientific, US), cleaned up with AMPure XP beads at 3:1 ratio (Beckman Coulter, US), and quantified by Quant-iT PicoGreen dsDNA Assay (Thermo Fisher Scientific, US). DNA Libraries were constructed from 1ng of genomic DNA with KAPA HyperPlus Kit (Roche, US) for FFPE DNA and amplified by 19 PCR cycles. Barcode adapters were used for multiplexed sequencing of libraries with SeqCap Adapter Kit A (Roche, US). Library size distribution was assessed on an Agilent 2100 Bioanalyzer using the DNA 1000 assay (Agilent, US), and its concentration was measured by Qubit® dsDNA HS Assay Kit (Thermo Fisher Scientific, US). Twelve samples were pooled at 18ng/ul and sequenced by Novogene (Sacramento, CA, USA) on 1 lane of the Illumina HiSeq Platform collecting 110G per 275M reads output of paired-end 150 bp read length. The median coverage per base is 2.0.

### Differentially expressed genes

We examined 6 HER2-positive patients that possess matched samples of both classes: DCIS_noamp and DCIS_amp. Two patients also have a few DCIS_int samples, which are excluded from the differential gene expression analysis. Since samples of patients 5 and 10 are sequenced in separate batches from the other 4 patients, to avoid potential batch effect, we applied batch effect correction on the raw dataset. Briefly, the dataset was normalized by variance stabilizing transformation in DESeq2 (version 1.26.0), and batch effect correction was performed using removeBatchEffect function from the limma package (version 3.42.2). Principal component analysis on the gene expression was performed using factoMiner (version 2.3) to assess the successful removal of batch effect in that samples are not clustered by batch but instead by other factors of interest. The batch-corrected dataset was used for differential gene expression analysis using the lme4 package (version 1.1.21) to fit the linear mixed model, in which “DCIS.group” is the fixed effect and “patient” is the random effect. P value was adjusted using Benjamini & Hochberg method. Differentially expressed genes at FDR <5% were identified. The differential gene expression analysis between DCIS_noamp and DCIS_neg was performed using DESeq2 (version 1.26.0). Differentially expressed genes were identified with log_2_foldchange > 0.75 and < −0.75 and padj < 0.05. Gene Set Enrichment Analysis (GSEA) (https://www.gsea-msigdb.org/gsea/index.jsp) and ToppGene Suite (https://toppgene.cchmc.org/) were used to perform the pathway enrichment analysis on the differentially expressed genes. To quantify the interferon pathway engagement for the identified interferon-stimulated gene (ISG) gene sets, an ISG signature score was generated using a rank-based statistic method by the singscore package (version 1.6.0).

### CNV analysis on Smart-3SEQ data and WGS data

To evaluate the cis-effect associated with HER2 amplification in Smart-3SEQ data, we inferred the copy number profiles at the HER2 locus and surrounding amplicon on chromosome 17 by averaging the relative gene expression over the 0.5Mb genomic regions [11]. Briefly, genes on chromosome 17 were aggregated in blocks of 0.5Mb by transcription termination site. The expressions in DCIS samples were normalized to the mean of normal breast samples and used to infer the copy number profiles. The inferred copy number profiles were visualized by heatmap.

To infer genome-wide CNVs for phylogeny reconstruction in both WGS data and Smart-3SEQ data, we first generated the bin-level log_2_ values using QDNAseq (version 1.22.0) on light-pass WGS data and CNVkit (version 0.9.5) on Smart-3SEQ data, respectively (Supplementary Figure 5). For the light-pass WGS data, duplicate reads were removed from bam files using picard and read counts per bin were calculated with the pre-calculated bin annotations for hg19 by QDNAseq and bin size 100 kbp. For Smart-3SEQ data, the bin-level log_2_ values were generated by normalization and bias-correction on the per-gene read counts using the CNVkit import-rna function. The following steps are the same for both data: once the bin-level log_2_ values are generated, multi-segmentation on a cohort of samples from each patient was performed using the copynumber package (version 1.26.0), so that common breakpoints across multiple samples can be found. Normal samples from each patient were used as their own reference. Since the LCM method renders a similar cellularity in all DCIS, we determined the absolute copy numbers uniformly by the following cutoffs: deletion = log_2_(0.75), loss = log_2_(0.875), gain = log_2_(1.125), amplification = log_2_(1.25). Focal CNVs that are less than 10 million bp were removed since many of them may be poorly called. Neighboring segments with the same CN aberrations were combined. An addition noise filtering step was performed to validate CNV calls inferred from Smart-3SEQ data: two sets of Gaussian noise data was generated by adding (1) mean of 0, sd of 1; and (2) mean of 0.5, sd of 1.5 to the original read counts. CNVs on the noise data were also called following the same steps above, and only CNVs that are called in at least 2 out of 3 datasets (1 original data + 2 noise data) will be called as the final CNVs. Due to the limited number of genes on small chromosomes (e.g., chr16-22) and high variance of the gene expression data, we only used CNV calls on chr1-15 to build the evolutionary trees and calculate the number of CNVs in each DCIS.

Since the sizes and boundaries of the segments called from Smart-3SEQ and WGS data are different, comparing CNVs directly between the two data would be challenging. Therefore, we extracted genes within each segment (on chr1-15) after the multi-segmentation step for both data and assigned the segment-level log_2_ values to individual genes. Spearman correlation coefficient was calculated at the gene-level log_2_ values.

### Phylogeny reconstruction on CNV calls

We used maximum parsimony to establish which samples shared common ancestors and therefore built phylogenetic trees using phangorn (version 2.5.5) and cellscape (version 1.10.0). To validate the tree structures, hierarchical clustering was performed on the identified CNVs and the significance of branches was assessed using the Pvclust package (version 2.2.0). The au (approximately unbiased) probability values (*p* values) are computed by multiscale bootstrap resampling, and clusters with au≥ 95% are considered strongly supported by the data.

## Results

### HER2-FISH analysis in DCIS

**Fig. 1:**
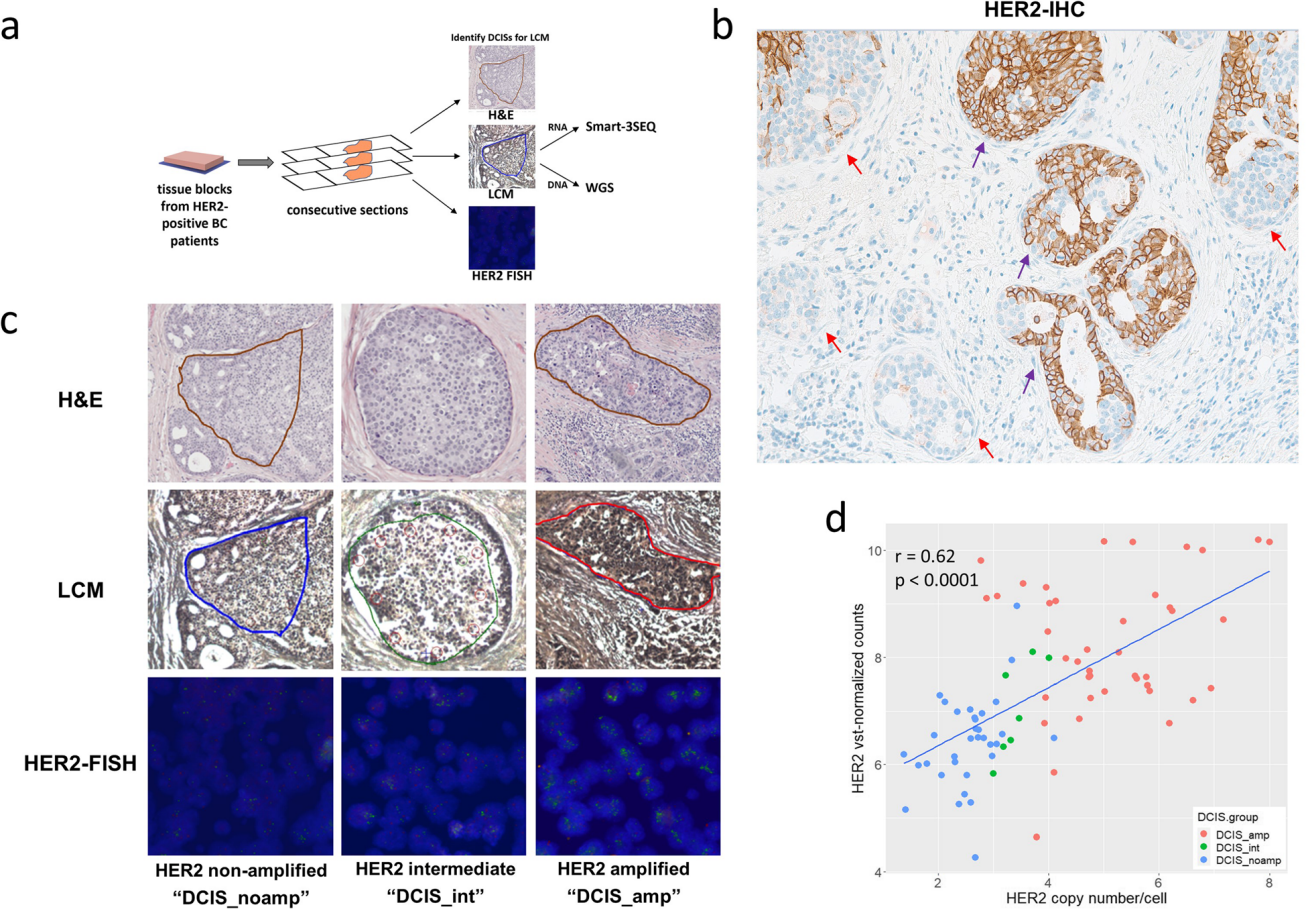
Tissue collection in DCIS with distinct HER2 status. **a** Workflow diagram for laser capture microdissection (LCM) Smart-3SEQ/WGS. Consecutive breast tissue sections were cut for H&E staining, LCM, and HER2-FISH. RNA and DNA were extracted from LCM-collected DCIS cells and subjected to Smart-3SEQ and WGS. **b** HER2-IHC staining for DCIS possessing different degrees of HER2 amplification with strongly stained ducts (purple arrows) and weakly stained ducts (orange arrows) at 200×. **c** Representative images of H&E (10×), LCM (10×), and HER2-FISH (400×) taken from consecutive sections of DCIS with different degrees of HER2 amplification. **d** Correlation between HER2 variance stabilizing transformation (VST)-normalized gene counts from Smart-3SEQ data and the HER2 copy number/cell from HER2-FISH results in DCIS. The HER2 copy number/cell is the absolute number of HER2-FISH signals/cell. It is calculated by averaging all detected cells in the DCIS duct

Here we employed spatial genomic methods that can quantify small amounts of RNA and DNA from single breast ducts isolated by laser capture microdissection (LCM)-collected formalin-fixed paraffin-embedded (FFPE) tissue [11] (Fig. 1a). This new approach enables us to profile multiple microscopic ducts that have different HER2 status within a single lesion, thus allowing us to uncover the evolutionary path during HER2 amplification in neoplasia progression. To evaluate the transcriptomic and genomic changes during HER2 amplification in DCIS, we screened 25 HER2-positive invasive breast cancer patients by HER2 immunohistochemistry (IHC) and selected cases with a large DCIS components. Among the 25 patients, all had HER2-overexpressing DCIS and 6 (24%) patients also showed multiple HER2-negative DCIS foci admixed with the HER2-overexpressing DCIS, indicating heterogeneity of HER2 status within DCIS in these patients (Fig. 1b). To quantify the degrees of HER2 amplification in these DCIS areas, we performed HER2-FISH on the 6 patients. This generates a map of HER2 status that allows us to profile the DCIS before and after HER2 amplification (Fig. 1c).

We cut tissue sections that are adjacent to the FISH sections and collected archival breast epithelial cells of individual DCIS ducts with distinct degrees of HER2 amplification using LCM. To quantify the HER2-FISH signals and in order to group the dissected ducts based on their HER2 status, we employed a deep learning-based image analysis approach that identified malignant breast epithelial cells and counted fluorescent signals inside the epithelial nuclear boundaries [12]. The DCIS regions were grouped into non-amplified or amplified (DCIS_noamp/DCIS_amp) groups, based on the probability distribution of their HER2 signals, which were obtained from all cells within the dissected regions. The comparison between the distributions showed that cells of DCIS_noamp have the highest probability at 1–3 signal/cell, whereas cells of DCIS_amp have a much wider distribution with a higher probability at ≥4 signal/cell (Supplementary Figure 2). A few ambiguous DCIS ducts were also observed with distributions between those of DCIS_noamp and DCIS_amp (Supplementary Figure 3) and were grouped as intermediate (DCIS_int). Principal Component Analysis on the probability distribution of HER2-FISH showed a separation between DCIS_noamp and DCIS_amp, while DCIS_int comprised an intermediate cluster (Supplementary Figure 4). We collected 93 samples (6 normal, 38 DCIS_noamp, 7 DCIS_int, 42 DCIS_amp) from a total of 6 patients (Supplementary Table 2). The age of patients ranges from 48 to 80 years. Four cases are estrogen receptor (ER)-positive, and three cases are progesterone receptor (PR)-positive. For all 6 patients, complete sets of matched normal, DCIS_noamp, and DCIS_amp were present. For two of the patients, multiple foci of DCIS_int were also observed and collected.

### HER2 amplification in DCIS alters transcriptome profiles and activates the interferon pathway

**Fig. 2:**
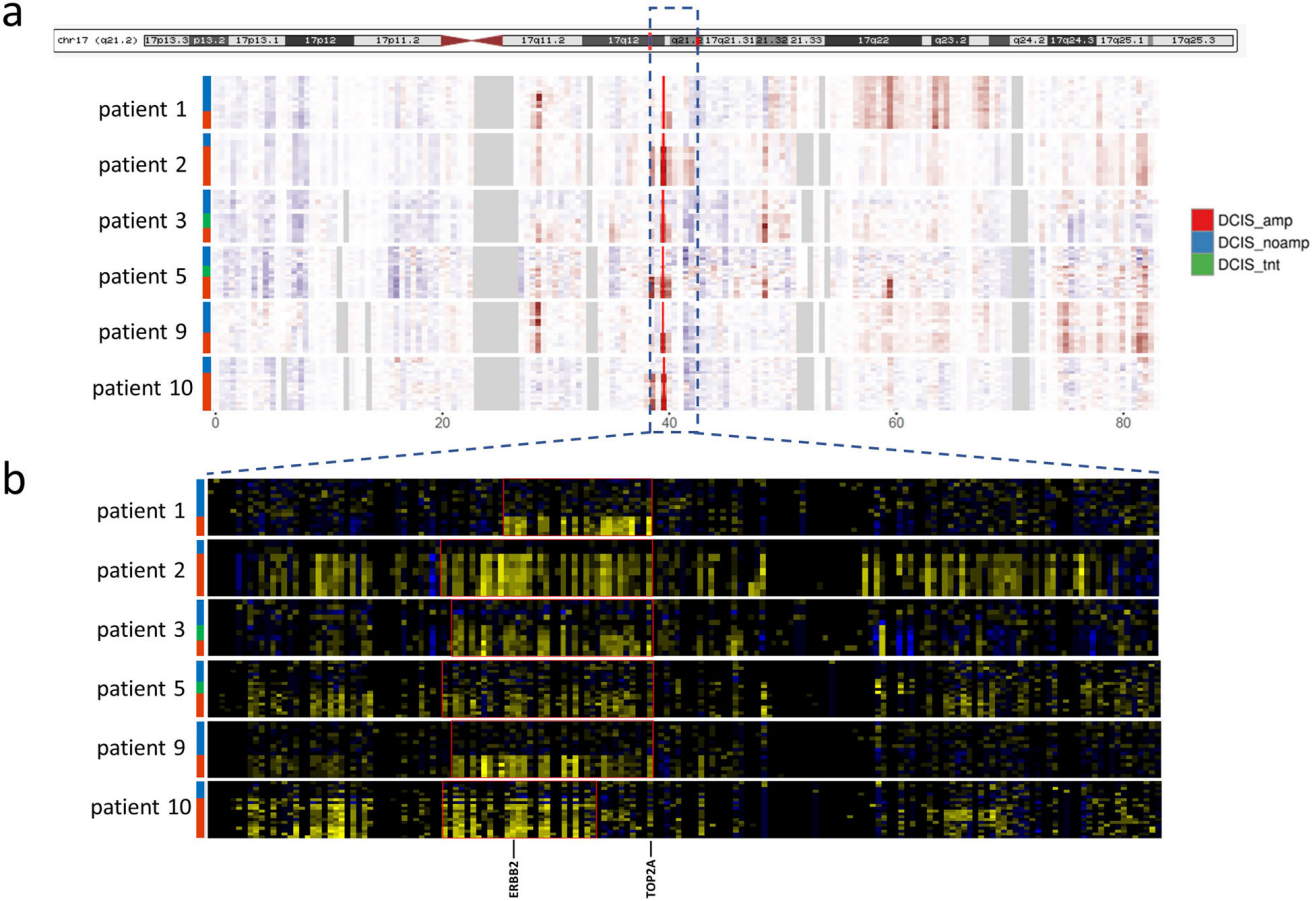
Cis-effect of HER2 amplification in DCIS from HER2-positive patients. **a** Inferred DNA copy number on chromosome 17 in DCIS. Genes are aggregated in blocks of 0.5Mb by transcription termination site. Heatmap cells show expressions normalized to the mean of normal breast samples. Red: higher expression than normal breasts; blue: lower expression than normal breasts; gray: no data. The blue-dashed box is where HER2 locus and surrounding amplicon locate. The bright red line shows the position of the HER2 locus. **b** Heatmap of relative expressions of genes in the blue-dashed box from 2a. Heatmap cells show expressions normalized to the means of matched normal breast samples. Yellow: higher expression than normal breasts; blue: lower expression than normal breasts. The red rectangle denotes the amplicon by visualizing the gene heatmap

Smart-3SEQ libraries were prepared from this collection of samples and directional next-generation sequencing yielded an average of 1.2 million uniquely mapped reads per sample. Using the Smart-3SEQ data, we first observed a correlation between HER2 normalized gene expression and the average HER2-FISH signal/cell per DCIS region (r = 0.62, p < 0.0001) (Fig. 1d), suggesting that HER2 overexpression in our samples is significantly correlated with HER2 amplification. To evaluate the cis-effects associated with HER2 amplification, we inferred the copy number profiles at the HER2 locus and surrounding amplicon by averaging relative expression levels over large genomic regions (Fig. 2a) [11]. While HER2 has been established as the key amplicon driver on chromosome 17q12 in HER2-amplified breast cancers, several other complex genomic aberrations on chromosome 17q have also been reported [14]. Indeed, the gene heatmap on chromosome 17q suggested that several genomic regions on 17q were recurrently amplified, including 17q11.2 and 17q21.32 (Fig. 2a). No recurrent amplifications were identified on chromosome 17p (Fig. 2a), which is consistent with previous report [14]. As described in previous literature [14], the HER2/TOP2A co-amplification was also frequent in our cohort (patients 1, 2, 3, 5, and 9) (Fig. 2b).

**Fig. 3:**
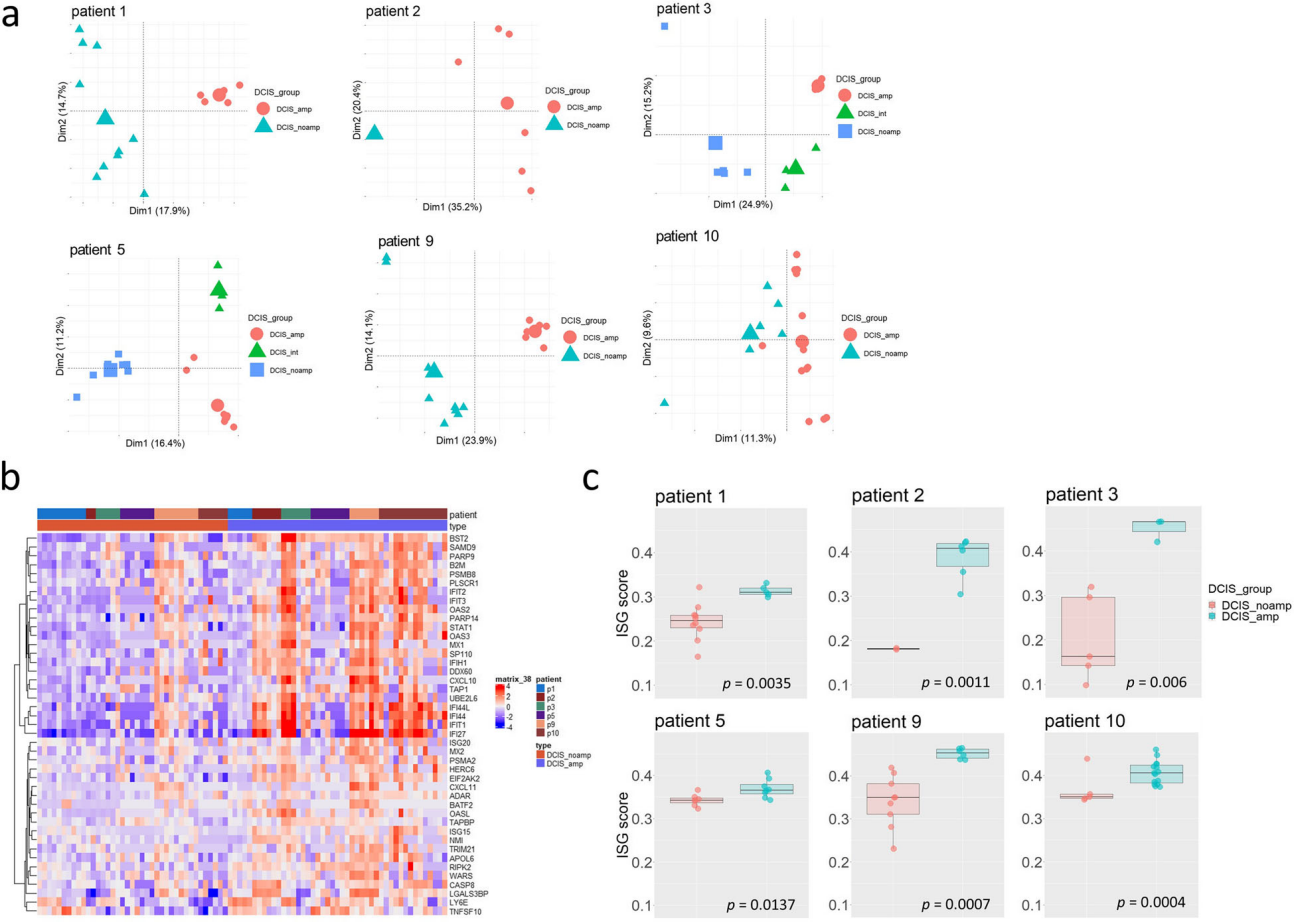
Trans-activating effect of HER2 amplification in DCIS from HER2-positive patients. **a** Principal component analysis on gene expression of DCISs for each patient. The samples were colored by their HER2 amplification status as measured by HER2-FISH. The larger symbols for each group denote the center of each cluster in PCA plot. **b** Heatmap of the mean-centered expressions of the 43 interferon-stimulated genes (ISGs) between DCIS_amp and DCIS_noamp. These 43 ISGs were identified by gene set enrichment analysis (GSEA) on the upregulated genes in comparison between DCIS_amp and DCIS_noamp. Patient IDs and sample types were denoted as the top bar. **c** Boxplot of ISG-signature scores between DCIS_amp and DCIS_noamp for each patient. The ISG scores are derived from the gene expressions of 43 ISGs

To assess the most variation in the transcriptomic profiles of DCIS, we selected the 2000 most highly expressed genes and performed PCA on the variance stabilizing transformation (VST)-normalized counts. The PCA from the gene expression data on combined DCISs of all HER2-positive patients showed that the patient explains the major difference in the transcriptomic profiles (Supplementary Figure 6), which implies the underlying heterogeneity of HER2-positive breast cancers. Patients 2 and 9 are clustered together, which might be due to their ER-negative status compared to other patients that are ER-positive (Supplementary Table 2). PCA for each patient showed that DCIS_noamp and DCIS_amp formed separate groups, while DCIS_int (patients 3 and 5) formed a third cluster (Fig. 3a), suggesting the existence of intermediate DCIS subpopulations during HER2 amplification.

To identify gene expression signatures associated with HER2 amplification in trans, we next assessed the differentially expressed genes between DCIS_noamp and DCIS_amp and performed gene set enrichment analysis against the identified genes with the gene set enrichment analysis (GSEA) and ToppGene analysis. Pathways involved in cell cycle and mitotic process, and others such as oxidative phosphorylation were enriched among the 683 genes upregulated in DCIS_amp (Supplementary Table 3); these pathways were also reported in a previous study using HER2-overexpressing vector in mammary cell cultures [15]. However, our study identified 43 genes encoding the interferon (IFN) signaling pathway as the most enriched gene family in DCIS with HER2 amplification (Supplementary Table 3, Fig. 3b); this pathway was not observed in the previous cell-based study [15]. A close examination on the 43 genes revealed that they belong to the interferon-stimulated genes (ISGs) [16, 17]. To further explore the expression profiles of the ISGs, we derived an ISG score using a 43-gene signature to quantify IFN pathway engagement. This analysis revealed a consistently higher ISG level in DCIS_amp (Fig. 3c), indicating that HER2 amplification activates the IFN signaling pathway in DCIS. To evaluate whether the IFN signaling activation is related to more inflammation, we reviewed areas of DCIS_noamp and DCIS_amp in all HER2+ patients and scored 0, 1, or 2 for no, little, or intense inflammation defined by the amount of lymphocytes present in the stroma for the overall area (Supplementary Table 4). It showed that 3/6 cases have more inflammation in DCIS_amp compared to DCIS_noamp. The inflammatory status does not seem correlated with the ISG score. In addition, we analyzed the ISG gene expression in a few HER2-amplified invasive samples (IDC_amp) from 5 patients (n=2 for p1, p2 and p5; n=1 for p3 and p10) (Supplementary Figure 7a). The ISG scores in IDC_amp are not different from that in DCIS_amp (*p* = 0.22) (Supplementary Figure 7b), indicating a consistent level of interferon signaling activation in the subsequent invasive cancer. To validate the association of increased IFN signaling with HER2 amplification, we also analyzed the ISG gene expression in a large cohort of HER2-positive breast cancer invasive carcinoma samples from The Cancer Genome Atlas (TCGA, 2015 Cell) [18] and found a significantly higher ISG score in 108 HER2-positive IDC samples compared to 644 HER2-negative IDC samples (supplementary Figure 8). These results indicated that the interferon pathway is activated by HER2 amplification in pre-invasive stage and this activation is persistent in the subsequent invasive cancer.

### HER2 amplification in DCIS increases diversity of copy number variations (CNVs)

Genome-wide DNA copy number variations (CNVs) have been increasingly recognized as a major cancer driver in various cancer types including breast cancer. Here we inferred CNV status using the gene expression data to study the genomic changes during DCIS progression. CNV prediction on gene expression data has been challenging due to the high variation of the data and gene-specific expression patterns. Previous methods were only suitable for visualization and were unable to predict the actual CNV calls that are needed for phylogeny reconstruction. Here we inferred the CNVs by integrating the CNVkit-RNA module [19] for bin file generation and the multi-sample breakpoint algorithm [20] for copy number segmentation. To validate the inferred CNV calls, we introduced a white Gaussian noise filtering step into the pipeline, so that only robust calls would remain (Supplementary Figure 5). The pipeline led to CNV inference at the chromosomal arm level. To validate the inferred CNVs on gene expression data, we collected several matched samples from consecutive sections and generated WGS data for CNV calling (Supplementary Figures 5 and 9a). The correlation coefficient of CNVs called between Smart-3SEQ and WGS data ranged from 0.42 to 0.72 with an average 0.57 (Supplementary Figure 9b).

**Fig. 4:**
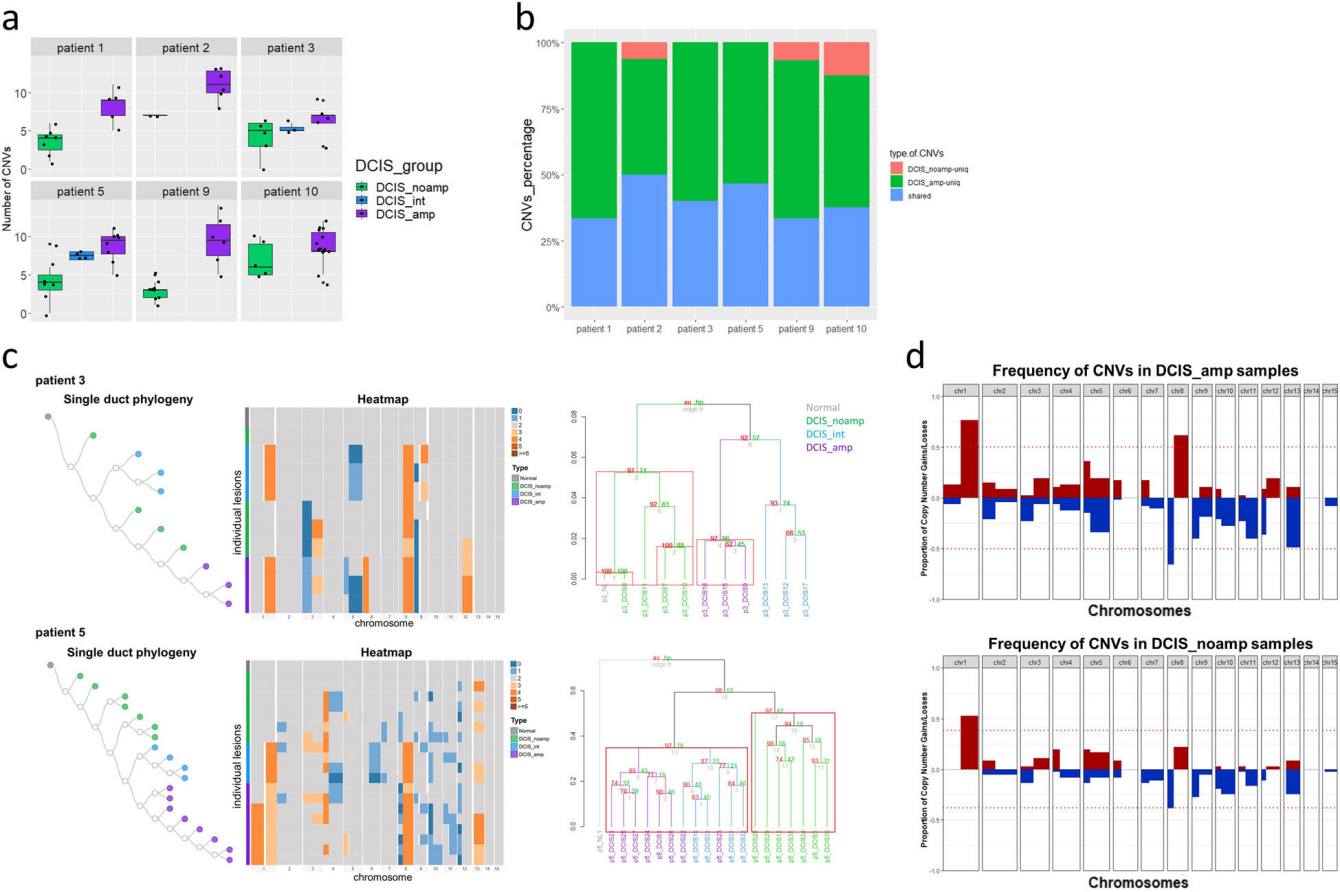
Evolutionary inference during HER2 amplification in DCIS from HER2-positive patients. We inferred copy number variations (CNVs) from Smart-3SEQ data and used CNVs called on chr1-15 for the following analysis. **a** Number of CNVs detected in DCIS with different HER2 amplification status. **b** Percentage of CNVs that are unique to DCIS_amp, unique to DCIS_noamp, or shared between two DCIS groups for each patient. A CNV is called if it is detected in ≥2 DCIS samples within each group. **c** Phylogenetic trees built by maximum parsimony (left) and hierarchical clustering (right) on inferred CNVs in DCIS for patients 3 and 5. Branches with the approximately unbiased p value (au) >=95% are considered statistically significant and highlighted by red rectangles (right). **d** Frequency of the CNVs detected among all patients by two DCIS groups along the chromosome location. Red: gain; blue: loss. The red dotted line denotes the value of (Q3+1.5×IQR) as the threshold. 1q gain, 8p loss, and 8q gain are called statistically significant in DCIS_amp; whereas 1q gain and 8p loss are called statistically significant in DCIS_noamp

The CNVs in DCIS were inferred individually since each patient represents an independent evolution. We first compared the chromosomal complexity among DCIS groups and found that in 5 out of 6 patients (patients 1, 2, 3, 5, 9), the average number of CNVs in DCIS_amp is higher than that in DCIS_noamp (Fig. 4a). DCIS_int from patient3 and 5 had intermediate number of CNVs (Fig. 4a). Within each patient, the CNVs were categorized into 3 categories: shared CNVs between DCIS_amp and DCIS_noamp, unique CNVs to DCIS_amp, and unique CNVs to DCIS_noamp. The number of shared CNVs together with the unique CNVs to DCIS_amp comprises 94% of the total CNVs (Fig. 4b), suggesting that DCIS_amp and DCIS_noamp share a common ancestor and particularly, DCIS_amp has more genomic aberrations than DCIS_noamp.

The CNVs shared between DCIS_amp and DCIS_noamp support a strong evolutionary relationship between these two subtypes. Cross-sectional DCIS heterogeneity provides a record of genomic alterations that occurred during DCIS progression into cancer, enabling phylogeny to be reconstructed by assuming that genomic complexity increases with time [21]. To delineate the clonal evolution during HER2 amplification in DCIS, we constructed phylogenetic trees from the inferred CNVs with maximum parsimony [22]. This analysis showed that DCIS_amp evolved from DCIS_noamp by acquiring new CNVs (Fig. 4c) (Supplementary Figure 10). Particularly, in two of the patients with DCIS_int collected (patients 3 and 5), DCIS_int and DCIS_amp formed two subclones that have distinct CNV events (Fig. 4c). For example, DCIS_amp in patient 3 had 3p loss and 12q gain whereas DCIS_int had 9q gain; DCIS_amp in patient 5 had 1p gain and 8p loss whereas DCIS_int had 6q loss. In addition, multiple subclones of DCIS_amp were observed in patient10 (Supplementary Figure 10). Hierarchical clustering of the inferred CNV profiles also identified multiple branches in patients 3, 5, and 10 (Fig. 4c) (Supplementary Figure 10). These results indicated that multiple events occurred for the acquisition of HER2 amplification in DCIS, and unique evolutionary paths existed in DCIS with different HER2 amplification status.

### DCIS acquires key CNVs prior to HER2 amplification

By counting the frequency of each CNV among all patients, we found that 1q gain (77%), 8p loss (66%), and 8q gain (62%) were the most frequent CNVs in DCIS_amp (Fig. 4d); whereas 1q gain (53%) and 8p loss (39%) were also among the most frequent changes occurred in DCIS_noamp (Fig. 4d). To explore whether MYC is an 8q gain precursor in our cohort, we checked its expression in DCIS and did not observe a significant difference between DCIS_noamp and DCIS_amp (Supplementary Figures 11a and 11b). We also collected HER2-amplified IDC from 5 patients and profiled their CNVs. Two examples of the phylogeny trees showed that IDC_amp shared a number of CNVs (especially 1q gain, 8p loss, and 8q gain) with their DCIS_amp/DCIS_noamp counterparts (Supplementary Figure 12). Importantly, IDC_amp was more clonally related to DCIS_amp compared to DCIS_noamp, consistent with the DCIS_amp lesions representing a later stage of DCIS, post HER2 amplification. Importantly, our findings suggest that 1q gain and 8p loss, two of the most recurrent CNVs in HER2-positive invasive breast carcinoma, can occur in DCIS even before the HER2 amplification has taken place. It is plausible that such initial genomic events (1q gain and 8p loss) render the HER2 amplicon area sensitive for subsequent amplification that give advantage to cell survival and proliferation.

To validate our findings in pure DCIS without invasive recurrences, we looked at the CNV changes in two previous studies using pure HER2-positive DCIS cohorts. Gorringe et al. [23] used the molecular inversion probe assay to profile CNVs in DCIS with and without recurrence, and they showed that 1q gain and 8p loss are among the most frequent CNVs in 8 HER2-positive non-recurrent DCIS and 9 HER2-positive recurrent DCIS. Another aCGH study by Waldman et al. [24] collected 5 HER2-positive initial DCIS samples (subject number F3, F11, F23, F29, F32). Among them, 4/5 acquired 1q gain and 3/5 acquired 8p loss, and these changes were also identified in their recurrent DCIS. In addition, we analyzed a subset of HER2-positive breast invasive carcinoma patients in breast invasive carcinoma dataset from TCGA [18]. The CNV profiles in HER2-positive cancers also showed that 1q gain, 8p loss, and 8q gain were among the most recurrent copy number changes (Supplementary Figure 13). Although the CNV profiles showed that 1q gain, 8p loss, and 8q gain are also common for breast invasive cancer as a whole (HER2-positive and HER2-negative) (Supplementary Figure 14), our findings are unique in that we observed that 1q gain and 8p loss can occur in DCIS even before HER2 amplification has taken place in HER2-positive breast cancer.

### Transcriptomic and CNV profiles in DCIS from HER2-negative breast cancer patients highlight the loss of extracellular matrix

**Fig. 5:**
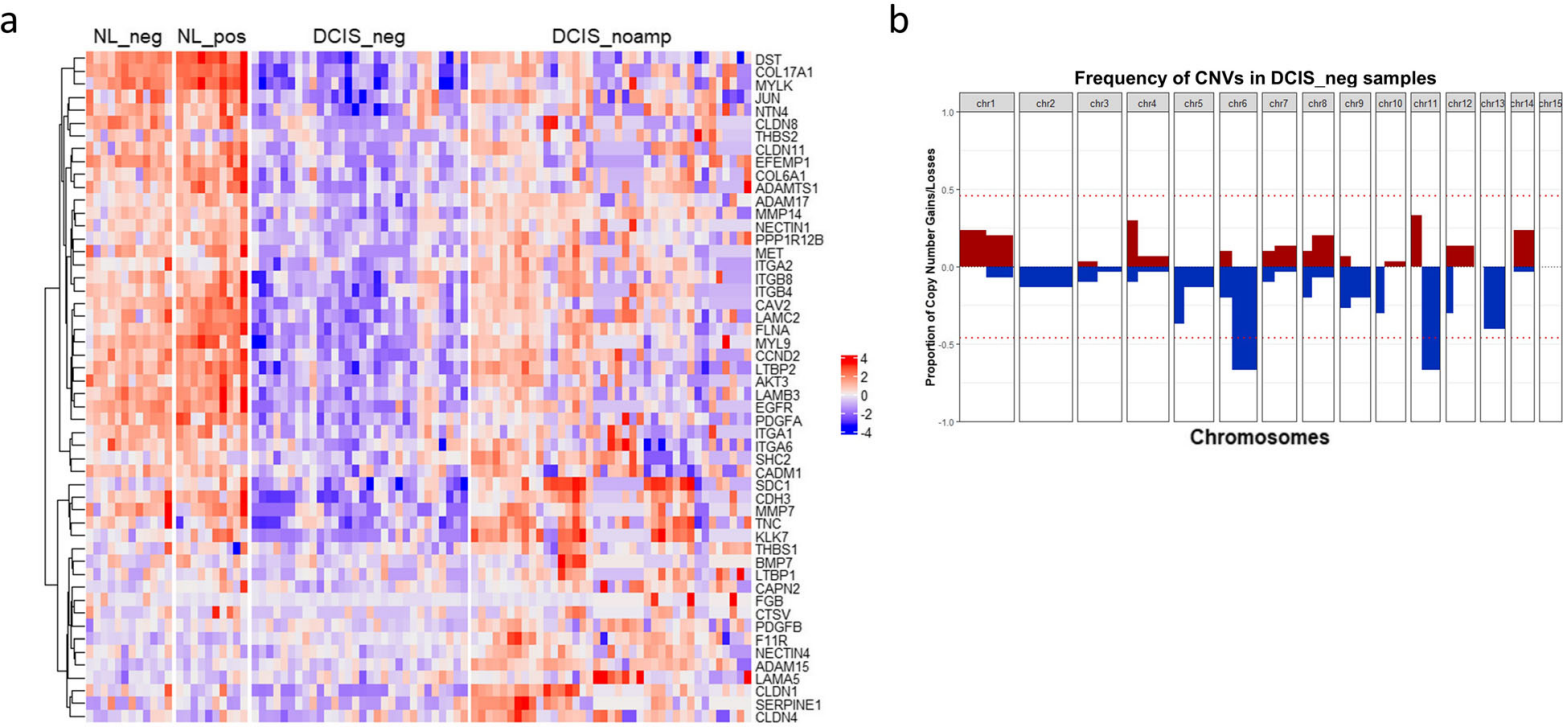
Transcriptomic and CNV profiles in DCIS_neg from HER2-negative patients. **a** Heatmap of the mean-centered expressions of genes involved in ECM organization and interaction in normal breast of HER2-negative patients (NL_neg), normal breast of HER2-positive patients (NL_pos), DCIS_neg, and DCIS_noamp. These enriched genes were identified by ToppFun analysis on the differentially up-regulated genes in comparison between DCIS_noamp and DCIS_neg. **b** Frequency of the CNVs detected in all DCIS_neg samples along the chromosome location. Red: gain; blue: loss. The red dotted line denotes the value of (Q3+1.5×IQR) as the threshold. 6q loss and 11q loss are called statistically significant in DCIS_neg

To assess the conditions that precede DCIS_noamp to becoming HER2-amplified, we collected 30 DCIS ducts (DCIS_neg) from 10 HER2-negative breast cancer patients (Supplementary Table 5). All these cases are ER-positive (Supplementary Table 5). In comparison to DCIS_noamp identified from HER2-positive breast cancer patients, DCIS_neg from HER2-negative breast cancer patients are also HER2 non-amplified, but they do not have the apparent potential to acquire HER2 amplification. We did not observe any DCIS_amp lesions in HER2-negative breast cancer patients. We compared the gene expression profiles of DCIS_noamp to DCIS_neg and observed 606 upregulated genes and 289 downregulated genes (Supplementary Table 6). ToppGene was used to perform pathway enrichment analysis, which showed that extracellular matrix (ECM) organization and interaction were the most overrepresented gene families in the comparison between DCIS_noamp and DCIS_neg (Supplementary Table 6). To better understand the expression pattern of the involved genes during DCIS progression, regions of normal breast from HER2-positive breast cancer patients (NL_pos) and HER2-negative breast cancer patients (NL_neg) were also compared (Fig. 5a). The comparison between DCIS_neg and NL_neg revealed a significant decrease in the expression of genes encoding proteins for ECM interaction (p < 0.0001) and PI3K-Akt signaling pathway (p < 0.0001). These include genes encoding integrins (ITGA2, ITGB8, ITGB4, ITGA1, ITGA6), laminins (LAMC2, LAMB3, LAMA5), growth factor receptors (EGFR), AKT3, MET, insulin receptor (INSR) and substrate (IRS1), and cyclins (CCND2, CDK6). Previous studies have indicated that ECM detachment occurs during tumor progression and cancer cells must develop resistance to anoikis (defined as the induction of cell death upon detachment from ECM) to survive in the absence of ECM [25]. Several mechanisms could contribute to cancer cell-developed anoikis resistance, including activation of pro-survival signals, growth factor receptor overexpression, or mutation/upregulation of key enzymes involved in integrin or growth factor receptor signaling [26]. We found that the above gene reductions observed in the comparison between DCIS_neg and NL_neg did not occur when NL_pos progressed into DCIS_noamp (Fig. 5a), suggesting that DCIS_noamp may have a higher ability to compensate for anoikis compared to DCIS_neg. Additionally, several in vitro studies using mammary epithelial cells demonstrated that HER2 can inhibit anoikis by compensating for the loss of EGFR and thereby evading BIM-EL-mediated anoikis [25]. Interestingly, a recovery in the loss of EGFR gene in DCIS_noamp that preceded HER2 amplification was also observed (Fig. 5a). These results suggest that the pre-existing compensation for the loss of ECM and EGFR may contribute to anoikis resistance that are important for priming cells for later acquisition of HER2 amplification for the development of HER2-positive cancer.

The distinction in the transcriptomic profiles between DCIS_noamp from HER2-positive patients and DCIS_neg from HER2-negative patients led us to suspect that the genomic structures from these two cohorts also differed. Indeed, the inferred CNV profiles in DCIS_neg from HER2-negative patients revealed that the most recurrent CNVs are 6q loss (67%) and 11q loss (67%) (Fig. 5b) (Supplementary Figure 15), which are different from that observed in DCIS_noamp from HER2-positive patients (1q gain and 8p loss) (Fig. 4c). This also suggested that compared to DCIS_neg that do not have the potential to acquire HER2 amplification in HER2-negative breast cancer, DCIS_noamp exhibit distinct genomic aberrations that may serve as a favorable structure for the later acquisition of HER2 amplification in HER2-positive breast cancer.

## Discussion

Most studies on human cancers involve advanced malignancies and do not examine the early changes that occur during oncogene activation. Although HER2 amplification is a common event that is seen in 15–20% of invasive breast cancers, it has been challenging to capture the biology at the stage that HER2 amplification occurs in pre-invasive breast neoplasia. This is largely due to the fact that tumors with recognizable areas before and after HER2 amplification are uncommon. More specifically, these in situ single-duct states are challenging to identify and capture by conventional RNA sequencing techniques. Smart-3SEQ, our newly developed RNA sequencing method on archival samples, allowed us to profile gene expression on specific FFPE regions of cells/ducts with known morphology and spatial context [11]. Using consecutive slides that are adjacent to the ones used for gene expression profiling, this method can be combined with other in situ methods for single duct analysis. This approach enables us to profile multiple microscopic ducts that have different HER2 status within a HER2-postive breast cancer patient, allowing us to uncover the biology and evolutionary path of HER2 amplification.

Our study reveals three important findings. First, the interferon pathway is activated by HER2 amplification in DCIS, which may provide a prolonged interferon signaling activation in HER2-positive breast cancer. Interferons are cytokines that play a critical role in both antiviral and antitumor immune responses [27–29]. However, accumulating evidence suggests that the magnitude and length of IFN signaling can differentially impact response to cancer therapies, for example by causing resistance to immune checkpoint blockade [30–33]. Our study observed an increased expression of IFN-stimulated gene (ISG) signatures after HER2 amplification, indicating an elevated IFN activation in DCIS harboring HER2 amplification. Previous studies demonstrated the existence of a wide variety of ISG signature-positive tumors that are associated with chronic activation of the IFN pathway [34]. This chronically activated state can be induced as part of the cellular response to DNA damage generated by genotoxic events such as genomic aberrations in cancer [35]. Our data demonstrates a higher genomic complexity as defined by increased number of CNVs in HER2-amplified DCIS compared to non-amplified DCIS. In addition, driver oncogenes such as HER2 are known to be frequently amplified on circular extrachromosomal DNA (ecDNA), which leads to the very high copy number that oncogenes can achieve in cancer [36–38]. These observations have led to the proposal that the genomic vulnerability caused by amplified HER2 on ecDNA and extra chromosome complexity associated with HER2 amplification, in the form of unrepaired DNA double-strand breaks, triggers chronic ISG signature in DCIS. In addition, HER2 amplification on ecDNA may affect transcriptional regulation differently from that amplified on linear structures, which may explain why IFN signaling was not induced in the promoter-driven HER2 overexpression system [15].

Second, the heterogeneity of HER2 copy number status in DCIS can be explained by their phylogenetic relationship where DCIS ducts with HER2 amplification evolve from surrounding ducts that lack HER2 amplification, and this evolutionary progression during the acquisition of HER2 amplification in DCIS involves multiple genomic changes. Evolutionary studies of breast cancer have focused on the inference of clonal evolution in primary tumor or the relationship of metastasis with the primary tumor [10, 39, 40]. However, recent studies have shown that most genomic aberrations evolve within the ducts prior to invasion [5, 9], raising the importance of delineating genomic evolution in early-stage breast cancer. Our study identified a large number of shared CNVs even prior to HER2 amplification, and additional CNV events that were acquired during HER2 amplification, suggesting a direct lineage relationship in the in situ components before and after HER2 amplification. In half of the patients, more than one genomic aberration event were present with acquired HER2 amplification in the in situ stage. This finding suggests that for the cases that are primed to amplify the HER2 locus, their pre-invasive ducts have a high propensity to acquire HER2 amplification rather than relying on purely random events. A future study with paired DCIS and invasive ductal carcinoma (IDC) will determine whether the multiple HER2-amplified DCIS clones will all be selected during invasion as shown in the “multiclonal invasion” model [9] or only the last clone be selected as suggested by the “clone selection” model [41]. Consistent with the findings that most genomic aberrations evolve within the ducts prior to invasion [9], our data show that most CNVs, including 1q gain, 8p loss, and 8q gain, were acquired within the ducts of the pre-invasive DCIS. The much higher genomic aberrations in HER2-amplified DCIS demonstrate that HER2 amplification is frequently associated with genomic rearrangements on multiple chromosomes [42]. More interestingly, we found that 1q gain and 8p loss were acquired in the ducts prior to HER2 amplification at the earliest stages of tumor progression. These observations can be explained in two aspects. First, from the standpoint of tumor biology, the genomic architecture defined by 1q gain and 8p loss may create a cellular state primed to acquire HER2 amplification. Second, genes in other areas particularly on 1q and 8p might collaborate with HER2 in the evolutionary advantage of the cells.

Third, pre-existing resistance to anoikis in pre-amplified DCIS may create a cellular state primed to HER2 amplification for growth advantage. Cancer cells must evade the ECM detachment-induced anoikis for tumor progression to occur. The study of how cancer cells develop anoikis resistance has been a subject of great interest in the past decade. Receptor tyrosine kinases such as growth factor receptors are one of the most commonly activated protein families in cancer cells and how these kinases contribute to tumor survival during ECM detachment is less understood. Previous studies using mammary epithelial cell culture discovered that the lack of ECM attachment caused diminished EGFR levels that correlated with an increase in the pro-apoptotic protein BIM, thus leading to anoikis induction [43]. Interestingly, HER2 overexpression can block anoikis by rescuing ECM detachment-induced deficiencies in EGFR and integrins [44, 45]. Indeed, our study found that during the progression from normal breast to DCIS in HER2-negative patients, expression of genes involved in ECM organization and interaction, as well as EGFR and several integrins are diminished. But interestingly, these losses are attenuated during the progression from normal breast to DCIS in HER2-positive patients. Furthermore, the losses of EGFR and others were prohibited even in the pre-amplified DCIS that are primed to but not yet becoming HER2-amplified. These results suggested that not only HER2 itself, but the cellular states that precede HER2 amplification in DCIS may restore the inhibition of anoikis by addressing ECM-detachment-induced deficiencies in EGFR and integrins. In addition, the pro-survival PI3K/AKT pathway can also be inhibited by ECM-detachment-induced deficiencies in EGFR and integrins [43]. Indeed, we observed deficiencies in the expressions of genes involved in PI3K/AKT pathway during DCIS progression in HER2-negative patients. This inhibition is again restored in the early-stage DCIS of HER2-positive patients. A thorough understanding of how the non-amplified DCIS restore the repertoire expression of the upstream regulators of anoikis, including cell-ECM interaction, integrins, EGFR, and PI3K/AKT pathway would be necessary.

Our cohort of mixed HER2 amplified and non-amplified DCIS is unique. We are the first to generate such a cohort to look at the gain of an oncogenic event within the context of DCIS. However, there are limitations to our study. Our major findings including the interferon signaling activation, ECM/EGFR changes, and CNV profiles need to be validated in a larger cohort. The small size of HER2-positive cases is due to the rarity of these samples. We screened ~50 HER2-positive cases and the vast majority demonstrated DCIS with just HER2 amplification. It is very uncommon to find an earlier precursor that is DCIS without HER2 amplification. Also, the intermediate DCIS stages with slightly amplified HER2 may be missed during our sub-sampling. A more delicate sub-sampling of DCISs during HER2 amplification will help us explore more details in this biological process.

## Conclusion

This study represents the first examination of the transcriptomic and genomic profiles during oncogene amplification in precancer lesions at a single-duct level. By using genomic methods that are spatially oriented, our study identifies several features that appear to generate insights into neoplastic progression at the oncogene level.

## Supplementary Information

**Additional file 1: Supplementary Figure 1.** Pipeline for automatic counting of FISH signals in DCIS epithelial cells. **Supplementary Figure 2.** The probability density of HER2-FISH signal/cell in DCIS_noamp and DCIS_amp references. The reference samples (18 non-amplified DCISs and 29 amplified DCISs) were handpicked from HER2-positive patients and the cells within each sample were combined into one reference for each group. The HER2-FISH signal/cell represents the absolute number of HER2-FISH signals in an epithelial cell in DCIS. The distribution of HER2-FISH signal/cell for all detected epithelial cells in a DCIS was represented by the probability density using kernel density estimation. **Supplementary Figure 3.** The probability distribution of HER2-FISH signal/cell in DCISs for each HER2-positive patient. The HER2-FISH signal/cell represents the absolute number of HER2-FISH signals in an epithelial cell in DCIS. The distribution of HER2-FISH signal/cell for all detected epithelial cells in a DCIS was represented by the probability density using kernel density estimation. For each patient, the density plots of HER2-FISH signal for each individual DCIS were overlayed into one plot. **Supplementary Figure 4.** Principal Component Analysis (PCA) on the distribution of HER2-FISH signal/cell in DCISs for each HER2-positive patient. The HER2-FISH signal/cell represents the absolute number of HER2-FISH signals in an epithelial cell in DCIS. The distribution of HER2-FISH signal/cell in a DCIS was represented by the probability density. **Supplementary Figure 5.** Pipeline of inferring genome-wide copy number variations (CNVs) from Smart-3SEQ and WGS data. **Supplementary Figure 6.** Principal Component Analysis (PCA) from gene expression data on combined DCISs of all HER2-positive patients showing that ‘patient’ factor explains the most variability. **Supplementary Figure 7.** (a) Heatmap of the mean-centered expressions of the 43 interferon-stimulated genes (ISGs) in DCIS_amp/DCIS_noamp and IDC_amp. These 43 ISGs were identified by gene set enrichment analysis on the up-regulated genes in comparison between DCIS_amp and DCIS_noamp. Patient IDs and sample types were denoted as the top bar. (b) Mean ISG signature scores in 5 patients with paired DCIS_amp and IDC_amp samples. The mean ISG score was calculated per case for DCIS_amp and IDC_amp separately if there are >1 samples in each group. Paired T-test was performed to determine the statistical significance between DCIS_amp and IDC_amp. p<0.05 is considered statistically significant. **Supplementary Figure 8.** Interferon signature gene (ISG)-signature scores between HER2+ and HER2- breast cancer invasive carcinoma patients from TCGA, 2015 Cell study. The ISG scores are derived from the gene expressions of 43 ISGs. **Supplementary Figure 9.** (a) Comparisons between CNVs inferred from Smart-3SEQ and WGS data in three HER2-positive patients (patient1, 3, 5) (red=gain, blue=loss). The brown rectangle box denotes CNVs on chr1-15, which were used to calculate the correlation between CNVs inferred from Smart-3SEQ and WGS data. (b) Spearman correlation coefficient on the gene-level log2 values between Smart-3SEQ and WGS data. **Supplementary Figure 10.** Phylogenetic trees built by maximum parsimony on inferred CNVs in DCIS for other 4 HER2-positive patients (patient1, 2, 9, 10). The CNVs were inferred from Smart-3SEQ data. **Supplementary Figure 11.** (a) Boxplot of the variance stabilizing transformation (VST)-normalized gene counts of MYC in DCIS_noamp, DCIS_int, and DCIS_amp from all HER2+ breast cancer patients. (b) Heatmap of the mean-centered expressions of MYC in DCIS_noamp, DCIS_int, and DCIS_amp. Patient IDs and sample types were denoted as the top bar. **Supplementary Figure 12.** (a) Summary of the HER2-amplified IDC samples in HER2-positive breast cancer patients. (b) Phylogenetic trees built by maximum parsimony on inferred CNVs in DCIS combined with their HER2-amplified IDC in patien5 and 10. The CNVs were inferred from Smart-3SEQ data. **Supplementary Figure 13.** Heatmap of copy number variations (CNVs) in 105 HER2-positive invasive breast cancer patients obtained from 2015 Cell, TCGA cohort (https://www.cancer.gov/tcga) (red=gain, blue=loss). Bars at the top indicate the ER-IHC, PR-IHC, HER2-IHC, and ERBB2 amplification status. Copy number data from HRE2-positive invasive breast carcinomas was obtained from the cBio Portal (https://www.cbioportal.org/). **Supplementary Figure 14.** Heatmap of copy number variations (CNVs) in 817 invasive breast cancer patients obtained from 2015 Cell, TCGA cohort (https://www.cancer.gov/tcga) (red=gain, blue=loss). Copy number data was obtained from the cBio Portal (https://www.cbioportal.org/). **Supplementary Figure 15.** Copy number variations (CNVs) inferred from Smart-3SEQ data in HER2-negative DCIS samples from 10 HER2-negative breast cancer patients (red=gain, blue=loss). CNVs called on chr1-15 were represented. 7 cases have 6q loss in DCIS_neg, and 8 cases have 11q loss in DCIS_neg. **Supplementary Table 1.** The posterior probability for a given cell with certain HER2-FISH signal being non-amplified/amplified. The probability distribution of HER2-FISH signal in amplified/non-amplified DCIS were determined by hand-picking the reference samples (18 non-amplified DCIS and 29 amplified DCIS) and combining them as one reference for each group. The posterior probability for a cell being HER2 non-amplified/amplified was calculated using the Bayesian formula, and the classification was assigned by the highest probability to which class the cell belongs to. **Supplementary Table 2.** Clinico-pathological information of 6 HER2-positive breast cancer patients. The left section showed patient ID, ER status and IHC intensity, PR status and IHC intensity, age, grade, and tumor size. The right section showed the number of samples for each patient, including normal breast, HER2 non-amplified DCIS, HER2 intermediate DCIS, and HER2 amplified DCIS. **Supplementary Table 4.** Inflammatory score in DCIS_noamp and DCIS_amp in HER2-positive breast cancer patients. Areas of DCIS_noamp and DCIS_amp in all HER2+ patients were reviewed and scored 0, 1, or 2 for no, little, or intense inflammation for the overall area. **Supplementary Table 5.** Clinico-pathological information of 10 HER2-negative breast cancer patients. The left section showed patient ID, ER status and IHC intensity, PR status and IHC intensity, age, grade, and tumor size. The right section showed the number of samples for each patient, including normal breast and HER2-negative DCIS.

**Additional file 2: Supplementary Table 3.** Differentially expressed genes in comparison between DCIS_noamp and DCIS_amp. Gene set enrichment analysis (GSEA) and ToppFun analysis results on the upregulated genes in DCIS_amp.

**Additional file 3: Supplementary Table 6.** Differentially expressed genes in comparison between DCIS_neg and DCIS_noamp. Gene set enrichment analysis (GSEA) and ToppFun analysis results on the upregulated genes in DCIS_noamp.

## Data Availability

The datasets used and/or analyzed during the current study are available from the corresponding author on reasonable request.
