# Supplementary material for: Transcriptome and genome evolution during HER2-amplified breast neoplasia
Source: Breast Cancer Res. 2021 Jul 15;23:73. doi: 10.1186/s13058-021-01451-6 (PMC8281634; doi:10.1186/s13058-021-01451-6)
Supplement: Supplementary file 1 — Additional file 1: Supplementary Figure 1. Pipeline for automatic counting of FISH signals in DCIS epithelial cells. Supplementary Figure 2. The probability density of HER2-FISH signal/cell in DCIS_noamp and DCIS_amp references. The reference samples (18 non-amplified DCISs and 29 amplified DCISs) were handpicked from HER2-positive patients and the cells within each sample were combined into one reference for each group. The HER2-FISH signal/cell represents the absolute number of HER2-FISH signals in an epithelial cell in DCIS. The distribution of HER2-FISH signal/cell for all detected epithelial cells in a DCIS was represented by the probability density using kernel density estimation. Supplementary Figure 3. The probability distribution of HER2-FISH signal/cell in DCISs for each HER2-positive patient. The HER2-FISH signal/cell represents the absolute number of HER2-FISH signals in an epithelial cell in DCIS. The distribution of HER2-FISH signal/cell for all detected epithelial cells in a DCIS was represented by the probability density using kernel density estimation. For each patient, the density plots of HER2-FISH signal for each individual DCIS were overlayed into one plot. Supplementary Figure 4. Principal Component Analysis (PCA) on the distribution of HER2-FISH signal/cell in DCISs for each HER2-positive patient. The HER2-FISH signal/cell represents the absolute number of HER2-FISH signals in an epithelial cell in DCIS. The distribution of HER2-FISH signal/cell in a DCIS was represented by the probability density. Supplementary Figure 5. Pipeline of inferring genome-wide copy number variations (CNVs) from Smart-3SEQ and WGS data. Supplementary Figure 6. Principal Component Analysis (PCA) from gene expression data on combined DCISs of all HER2-positive patients showing that ‘patient’ factor explains the most variability. Supplementary Figure 7. (a) Heatmap of the mean-centered expressions of the 43 interferon-stimulated genes (ISGs) in DCIS_amp/DCIS_noamp and [file 13058_2021_1451_MOESM1_ESM.pdf]

Supplementary Figure 1

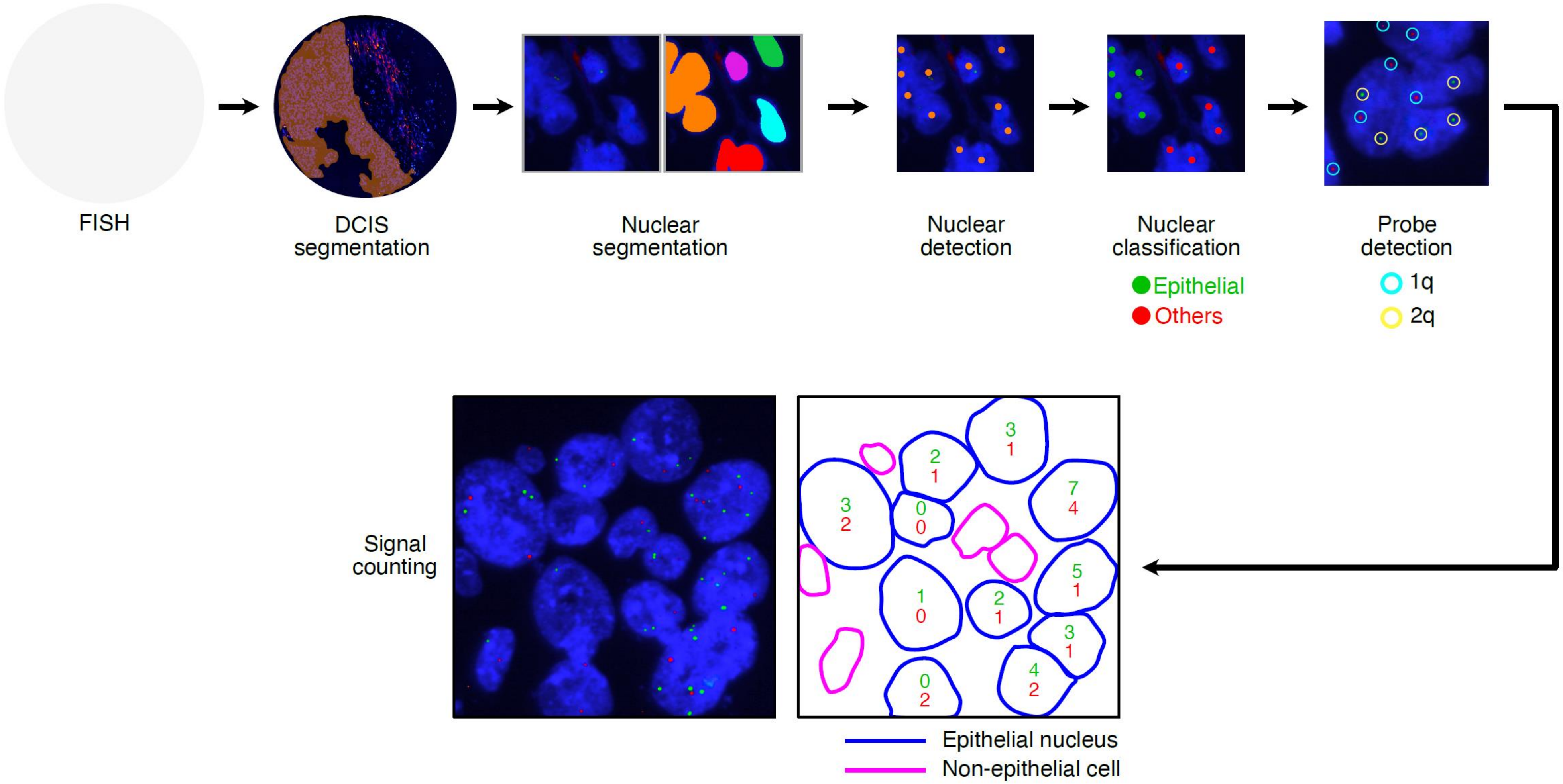

Supplementary Figure 2

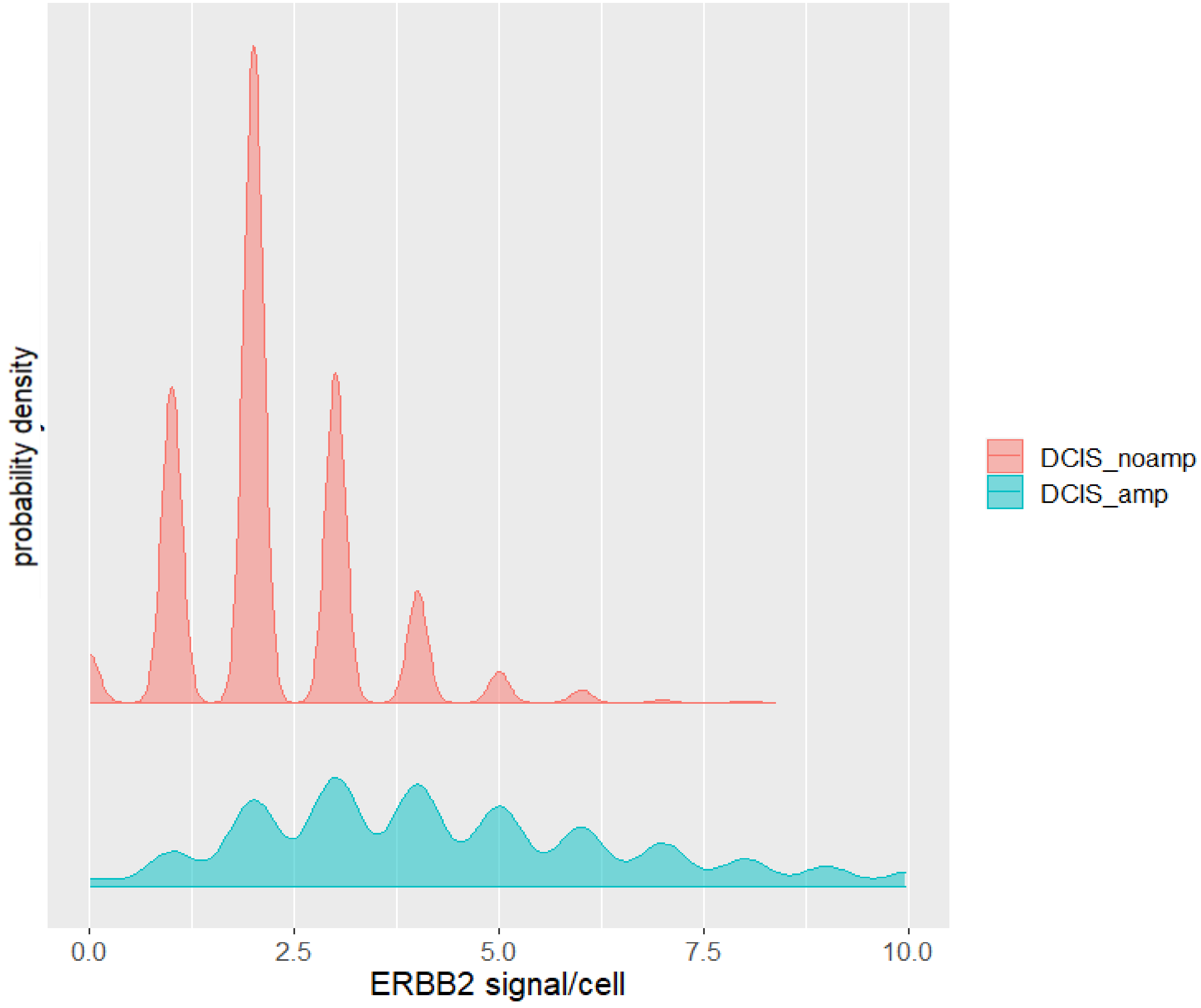

Supplementary Figure 3

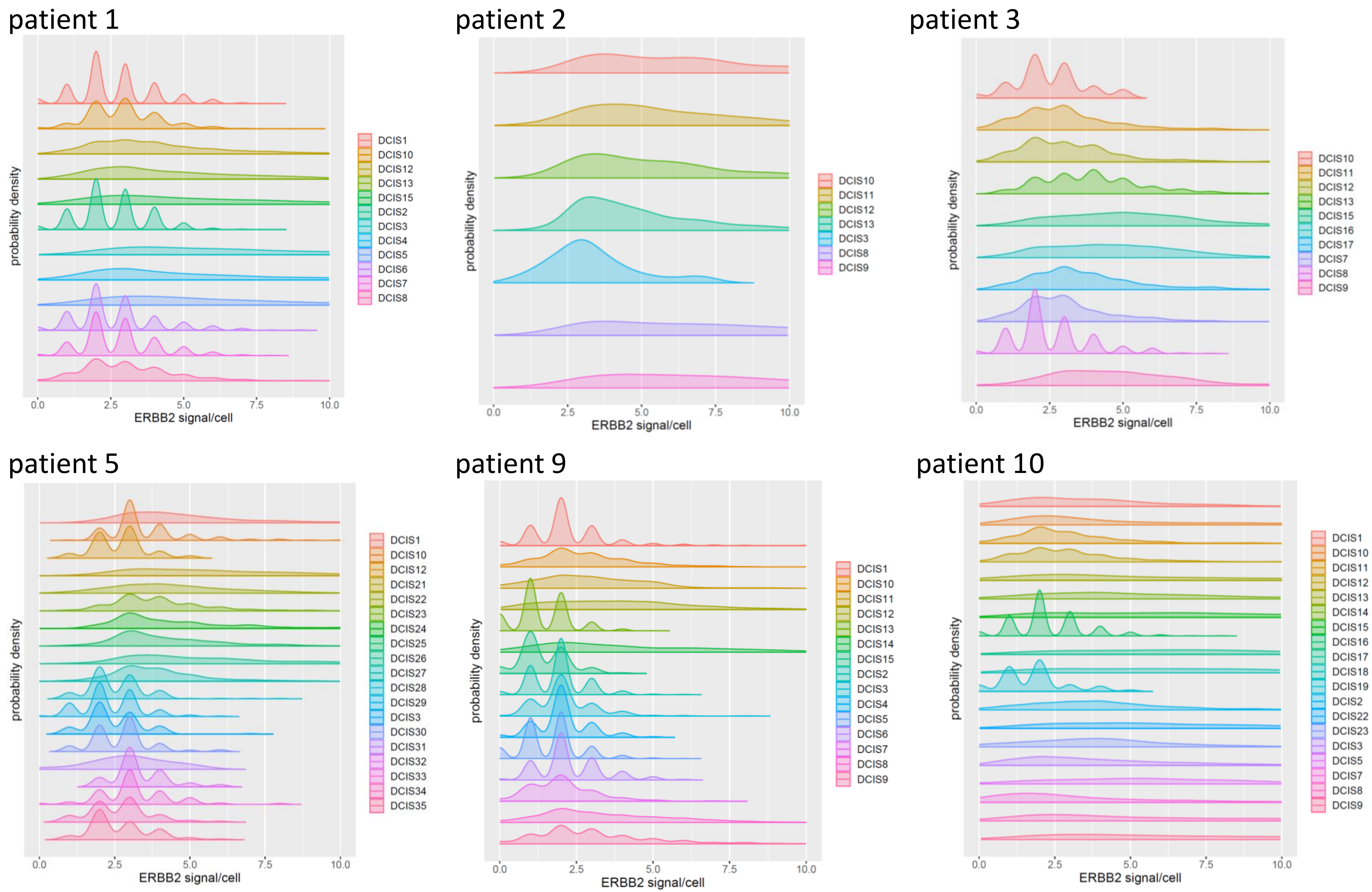

Supplementary Figure 4

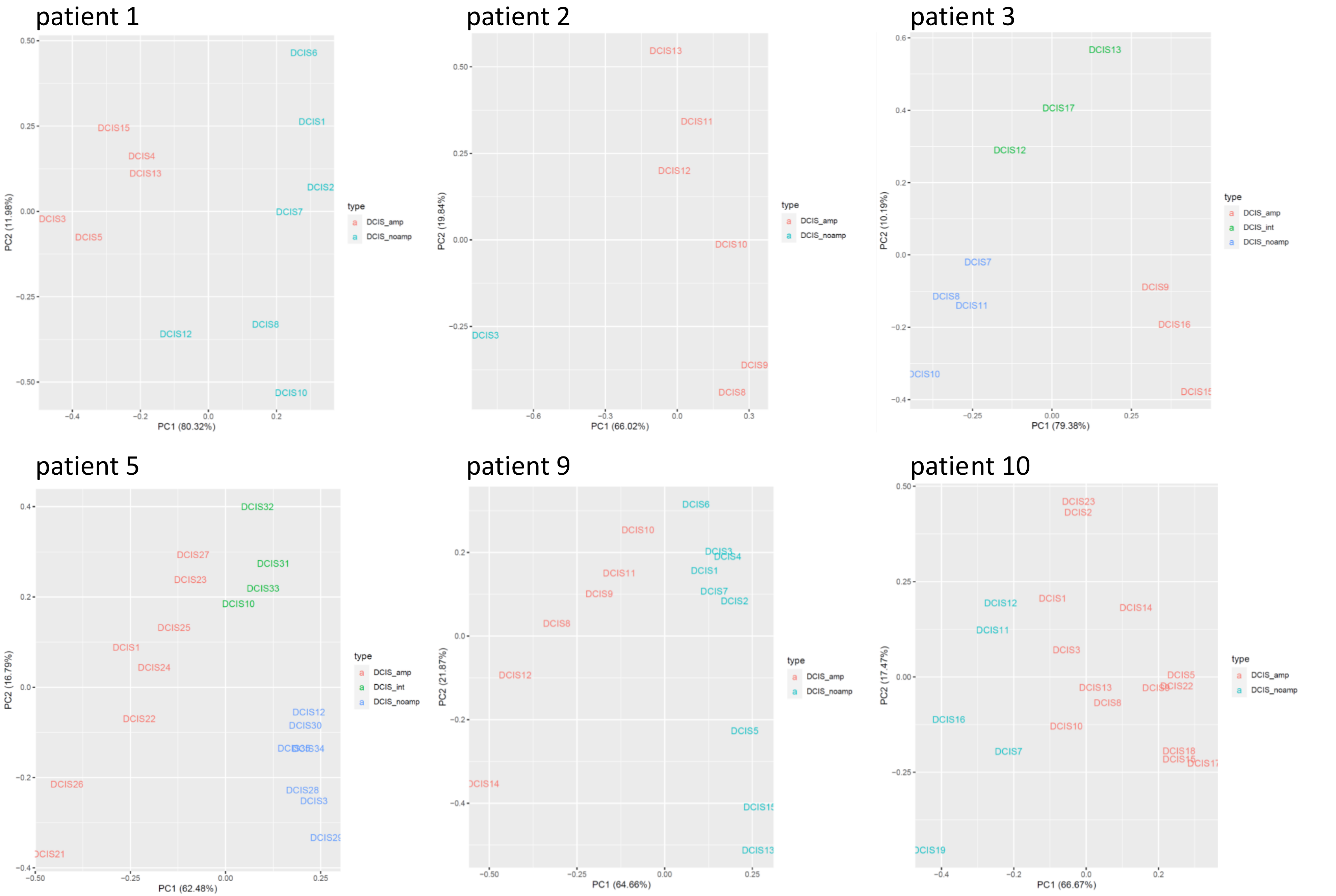

Supplementary Figure 5

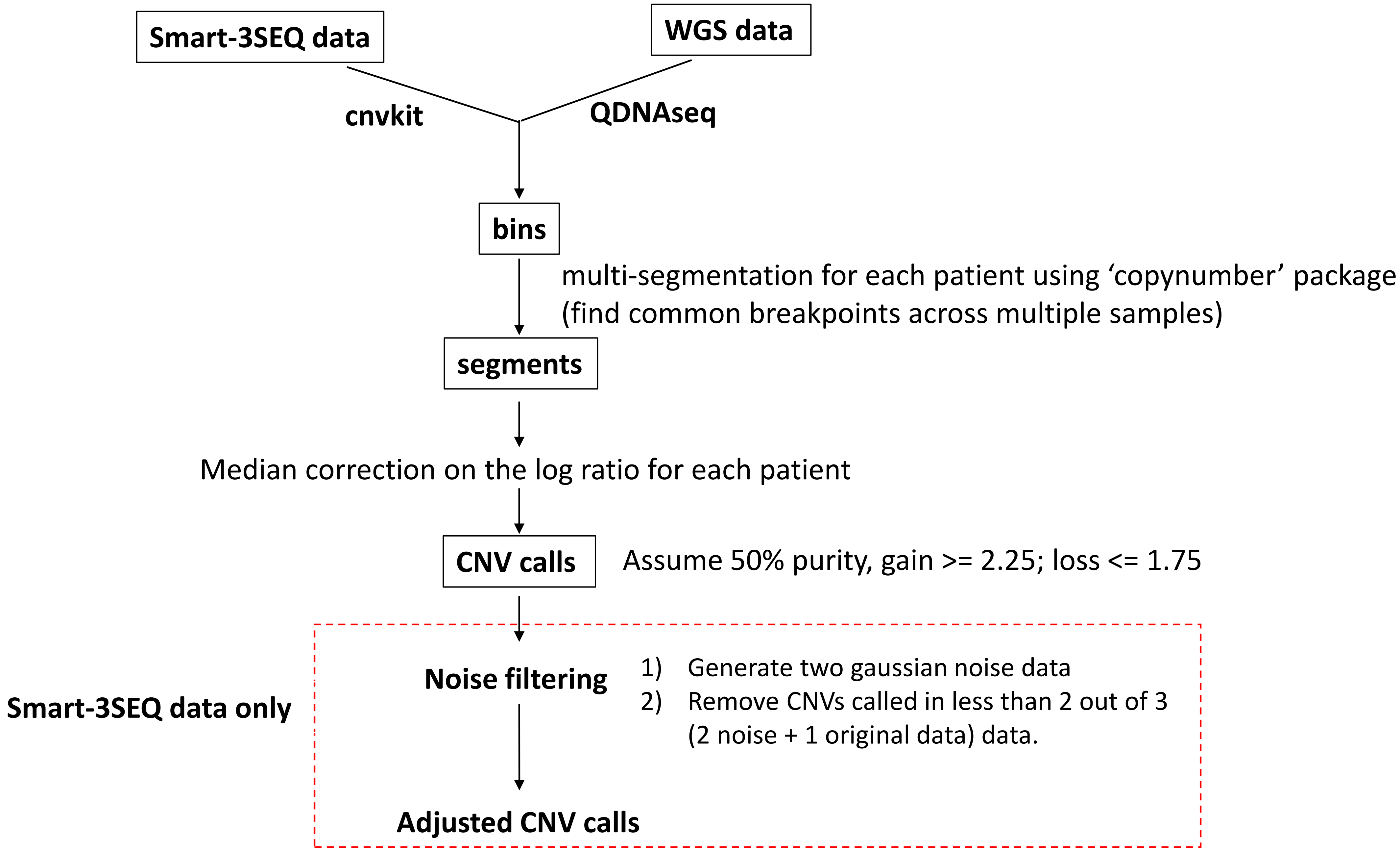

Supplementary Figure 6

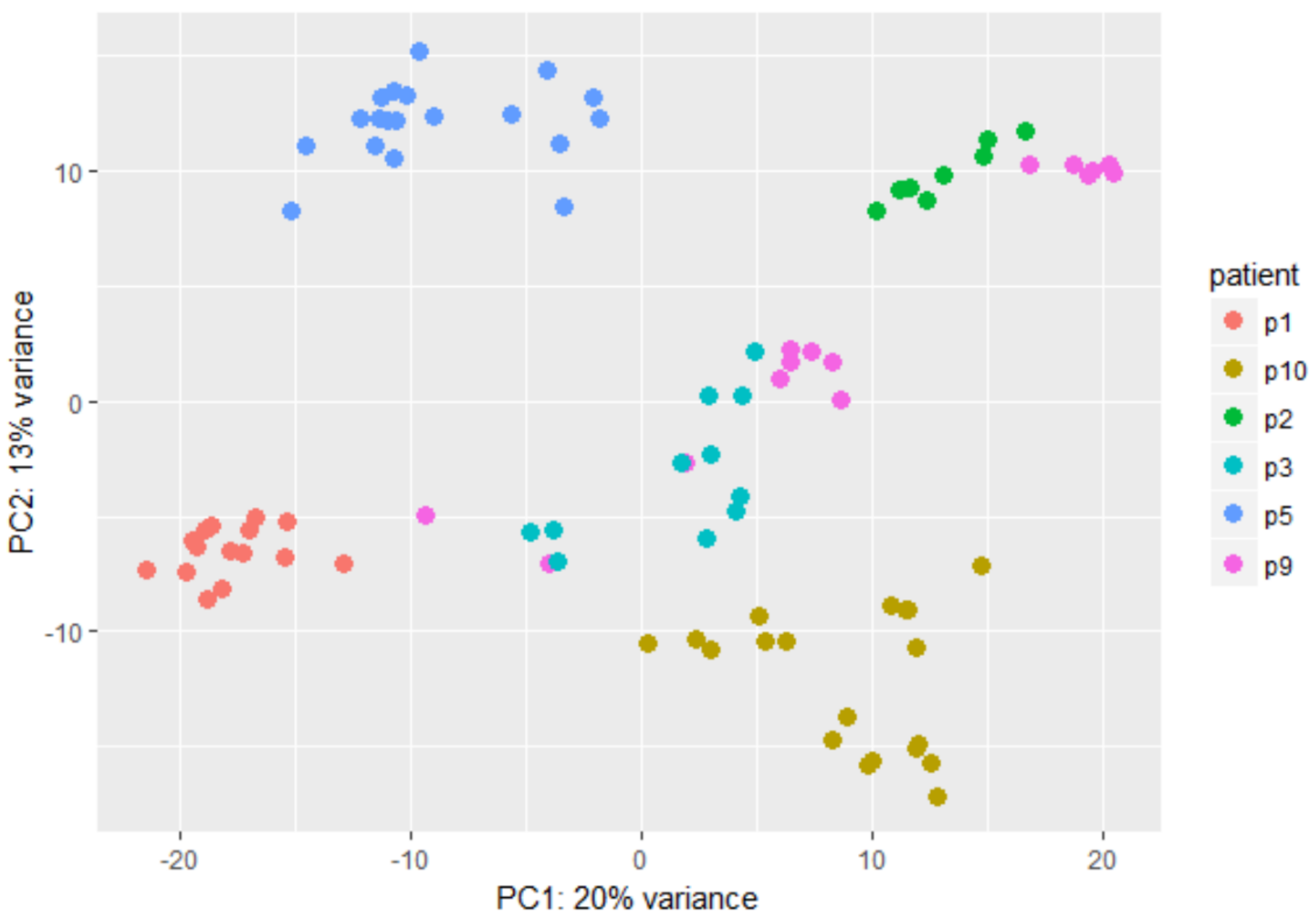

Supplementary Figure 7

a

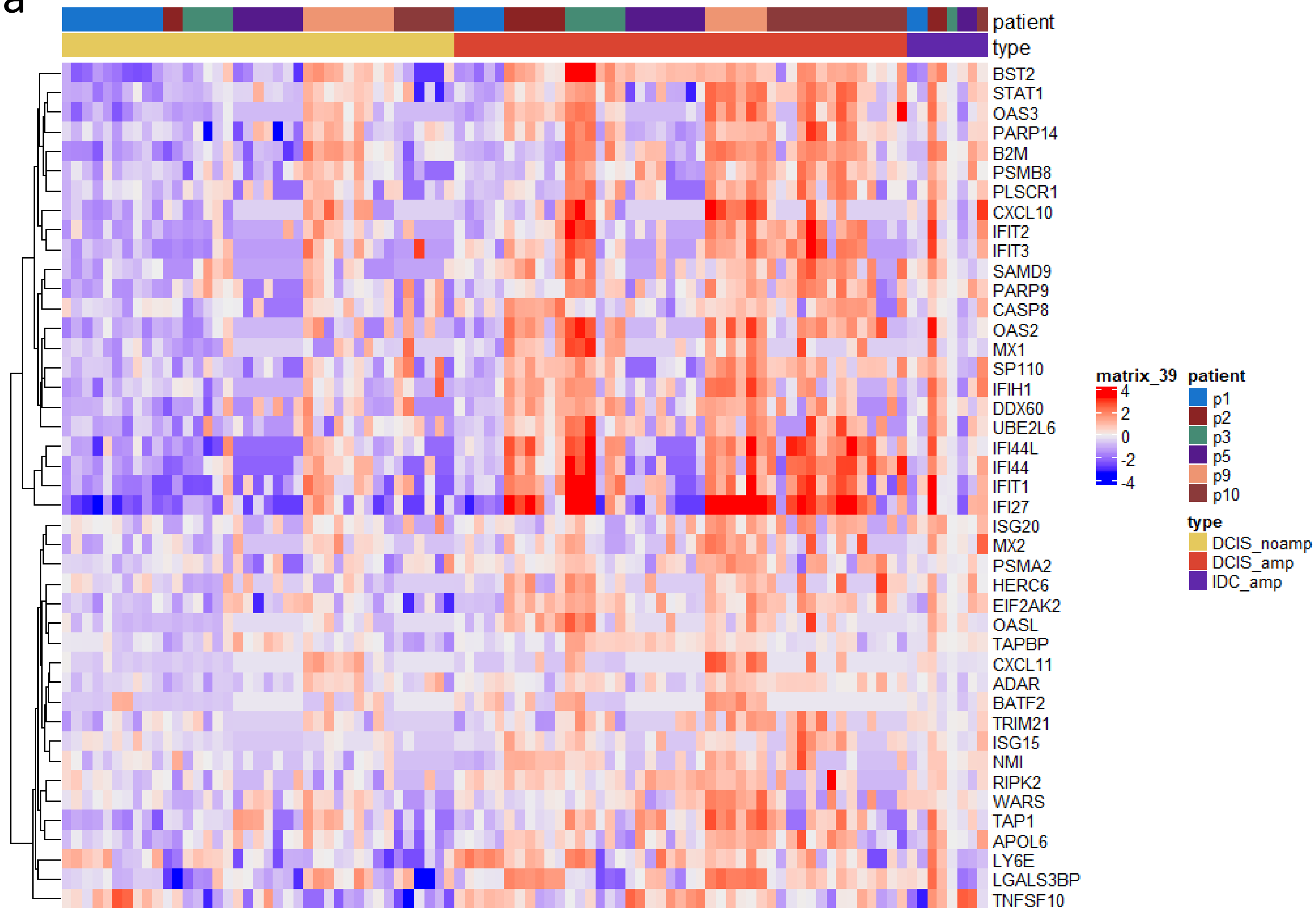

b

patient.id	DCIS_amp	IDC_amp
p1	0.258384	0.382353
p2	0.365297	0.434547
p3	0.441625	0.418409
p5	0.367363	0.39182
p10	0.413918	0.437682

Supplementary Figure 8

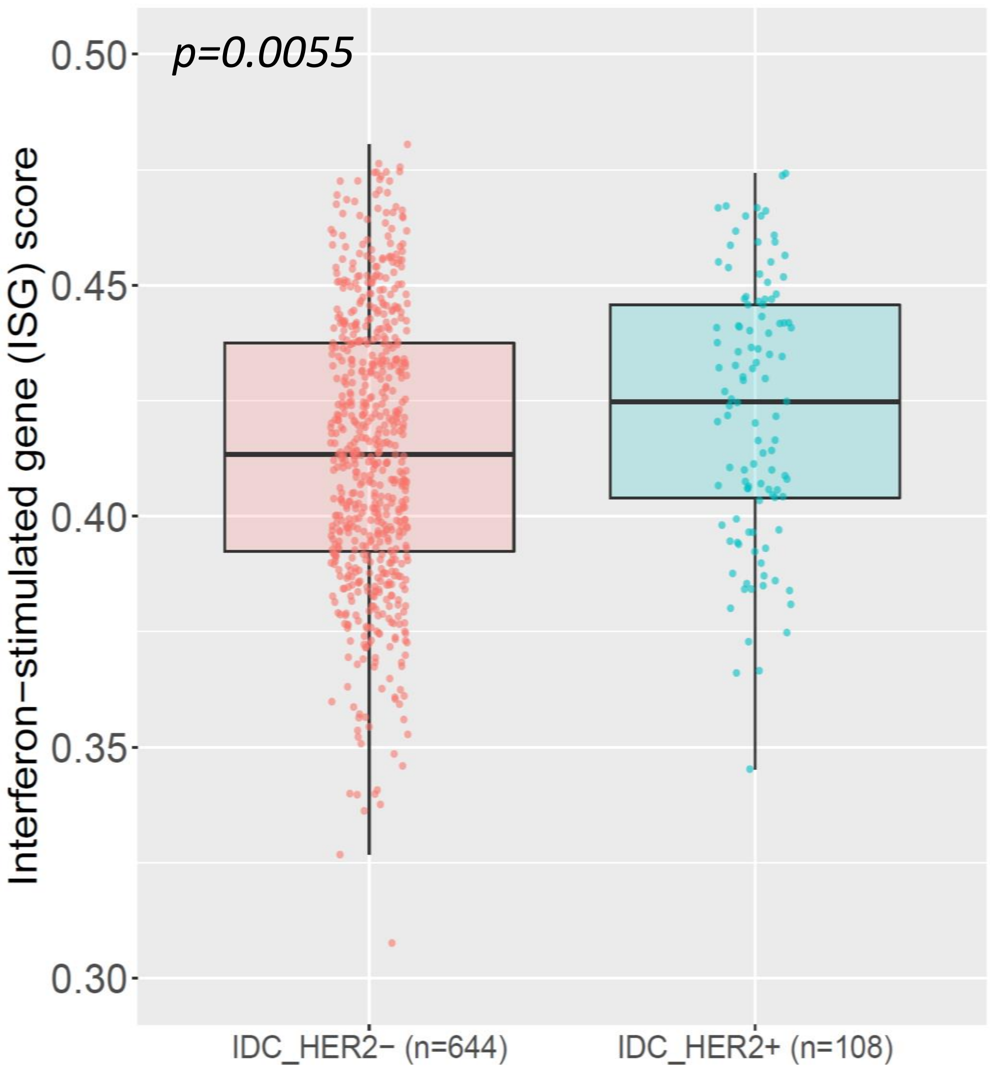

Supplementary Figure 9

a

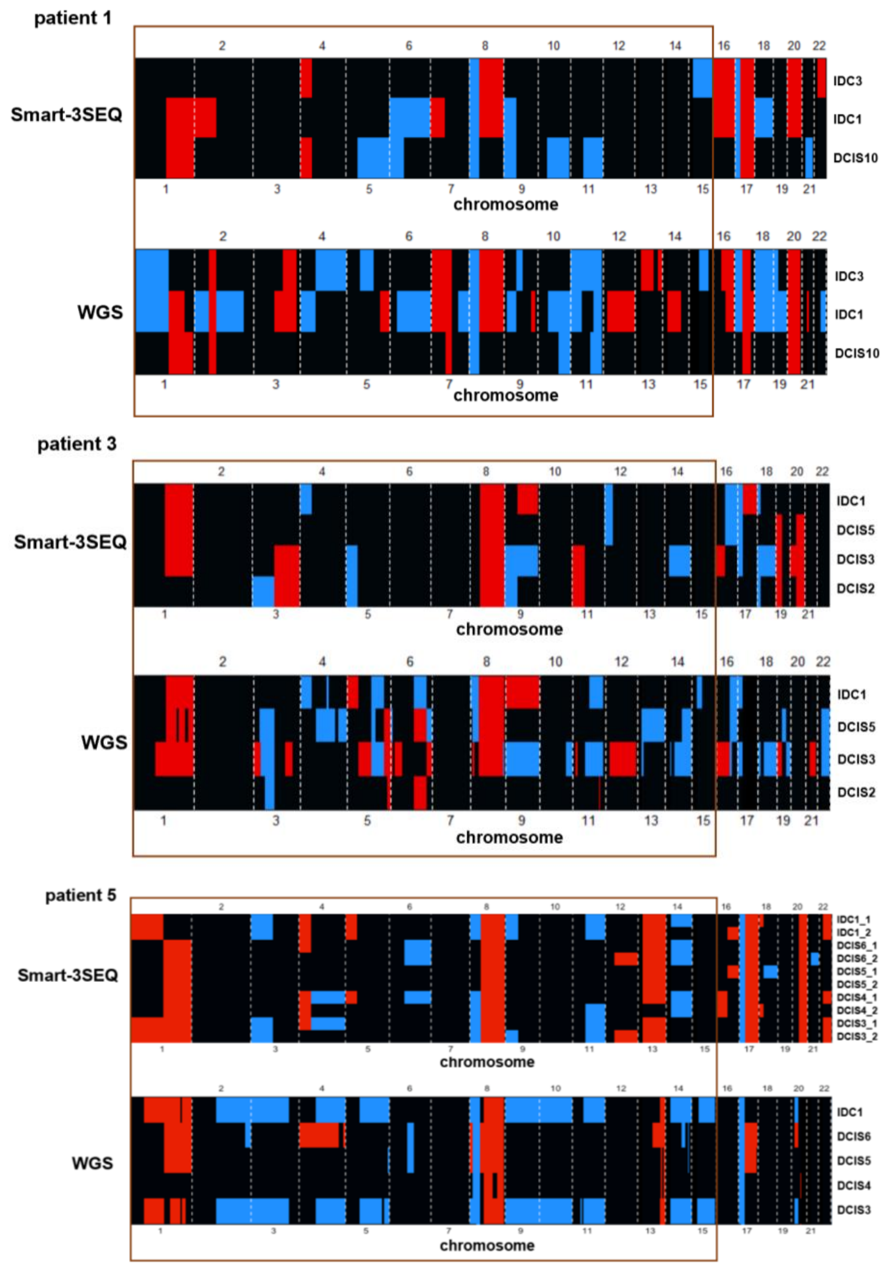

b

Sample	Spearman correlation coefficient of the gene-level log2 value between Smart-3SEQ and WGS data
p1_DCIS10	0.718
p1_IDC1	0.684
p1_IDC3	0.506
p3_DCIS2	0.503
p3_DCIS3	0.561
p3_DCIS5	0.526
p3_IDC1	0.54
p5_DCIS3	0.647
p5_DCIS4	0.497
p5_DCIS5	0.631
p5_DCSI6	0.417
p5_IDC1	0.597

Supplementary Figure 10

patient 1

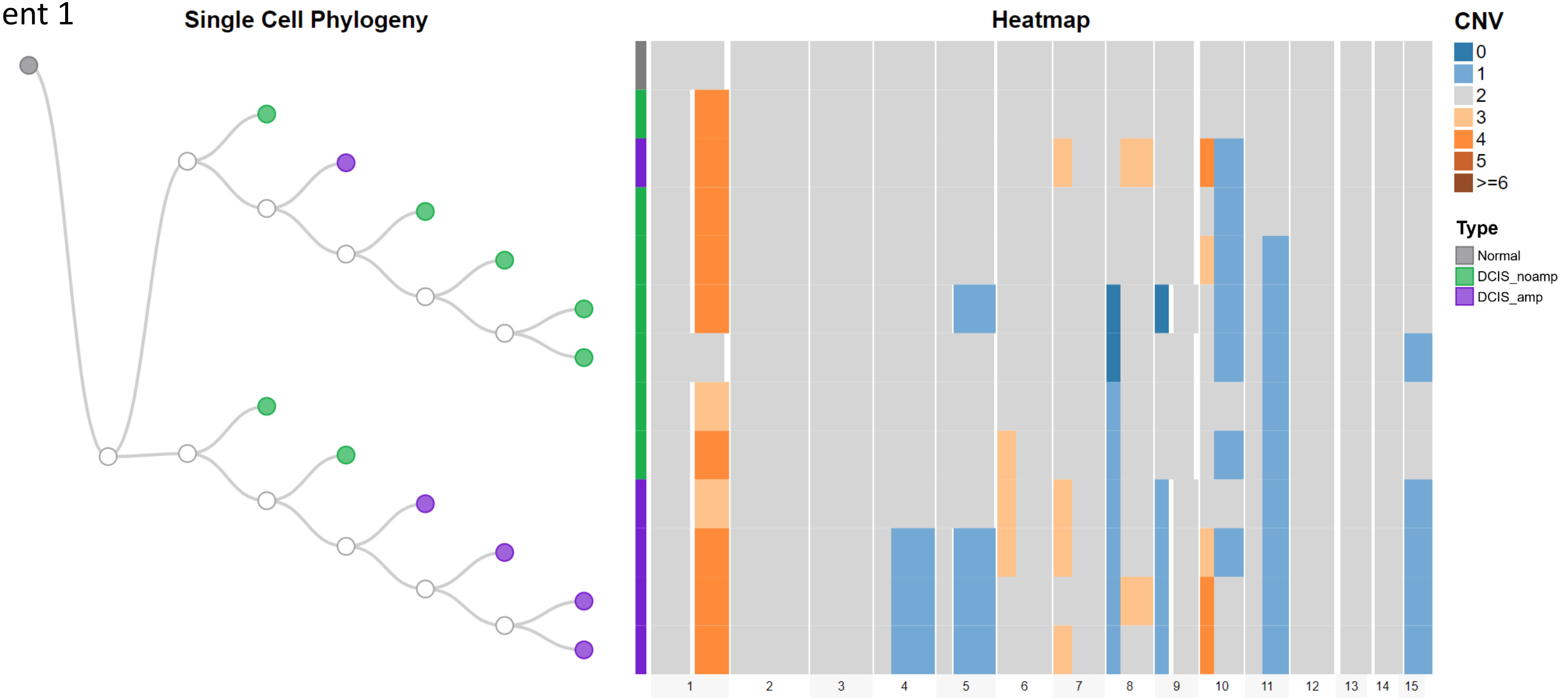

Supplementary Figure 10

patient 2

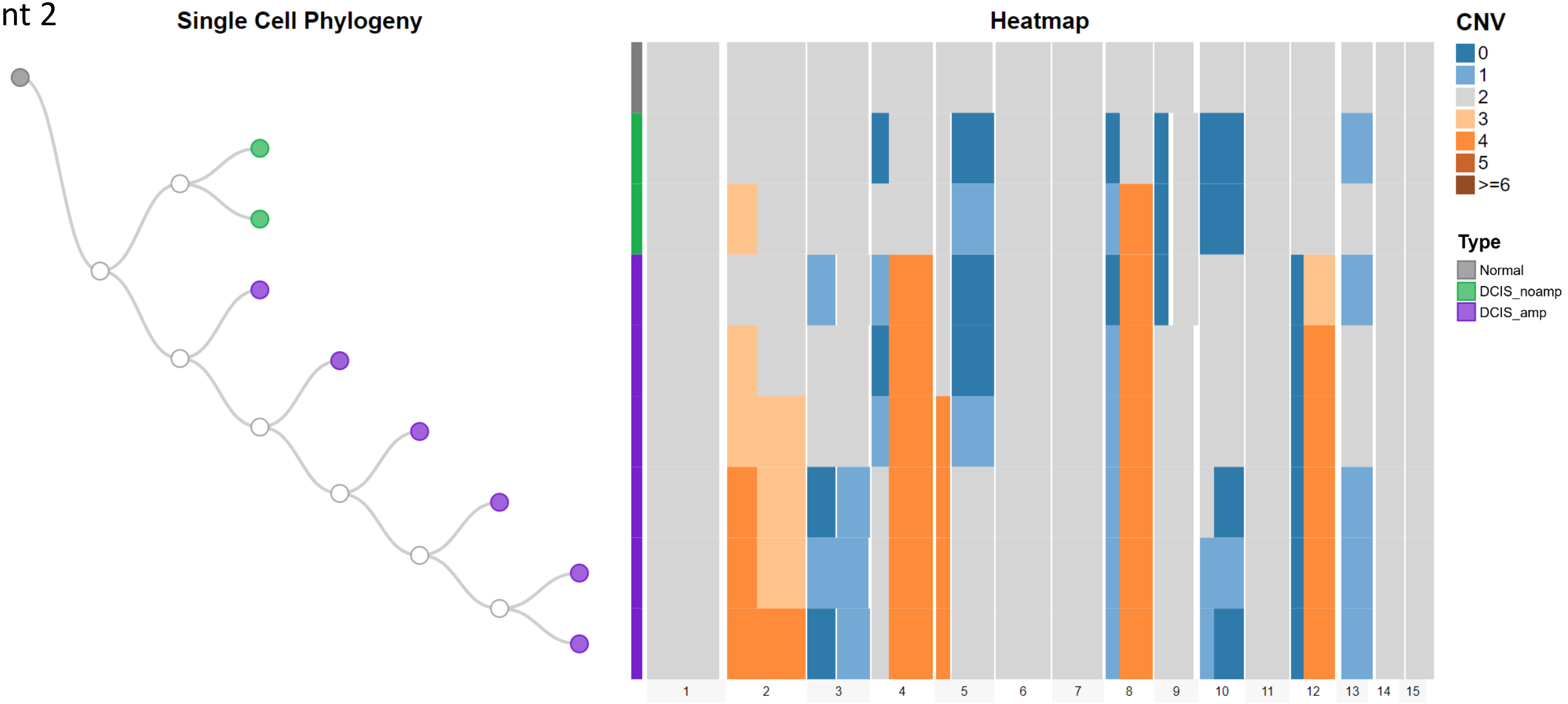

Supplementary Figure 10

patient 9

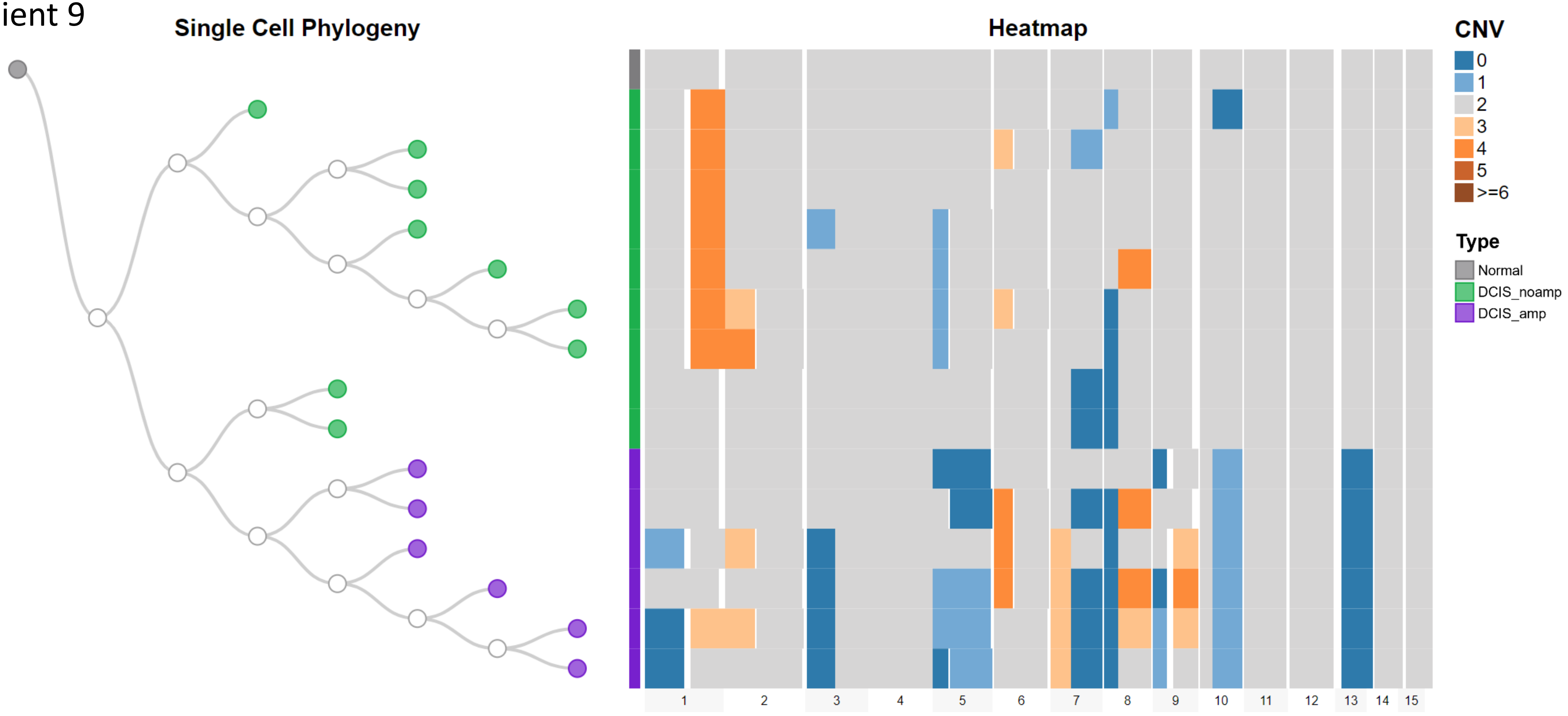

Supplementary Figure 10

patient 10

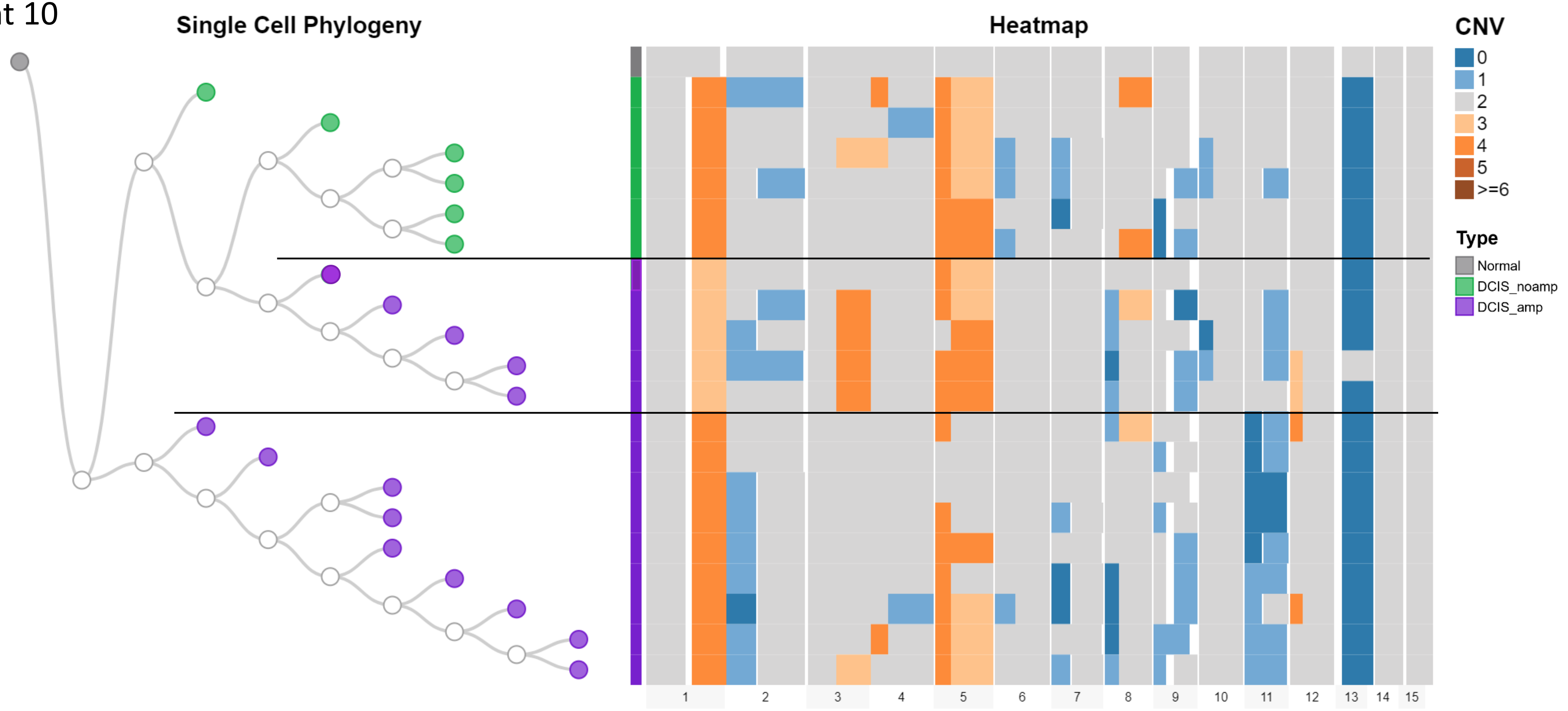

Supplementary Figure 11

a

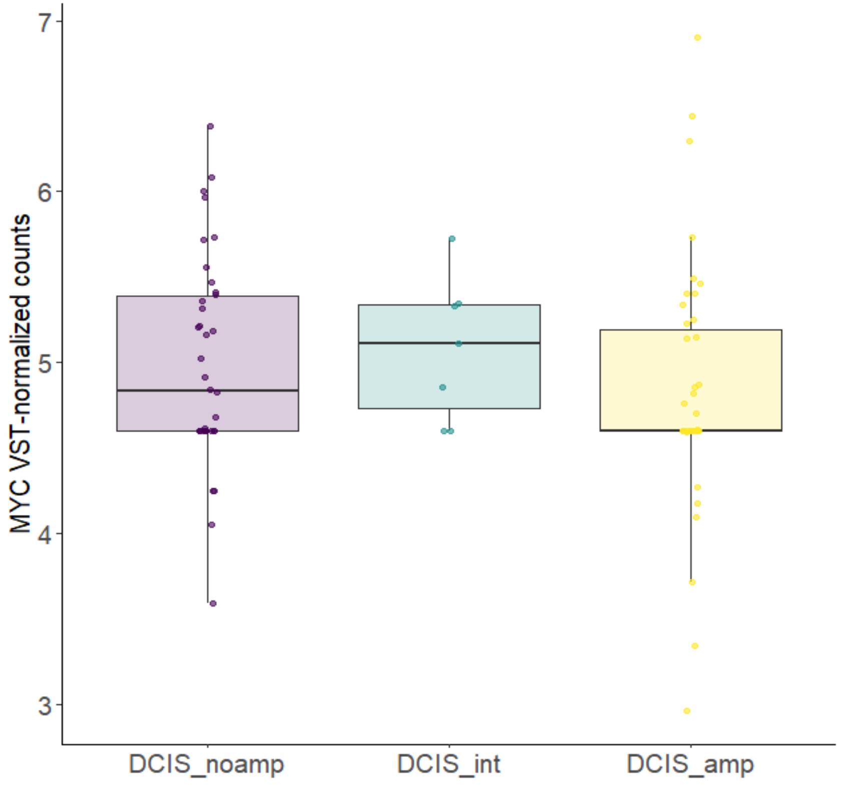

b

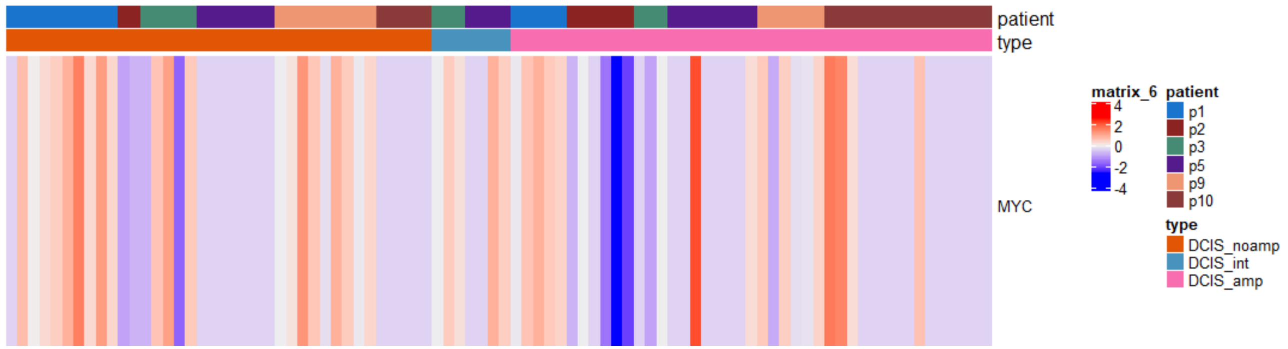

Supplementary Figure 12

a

patient	number of IDC samples
p1	1
p2	2
p3	1
p5	2
p9	0
p10	1

b

patient 5

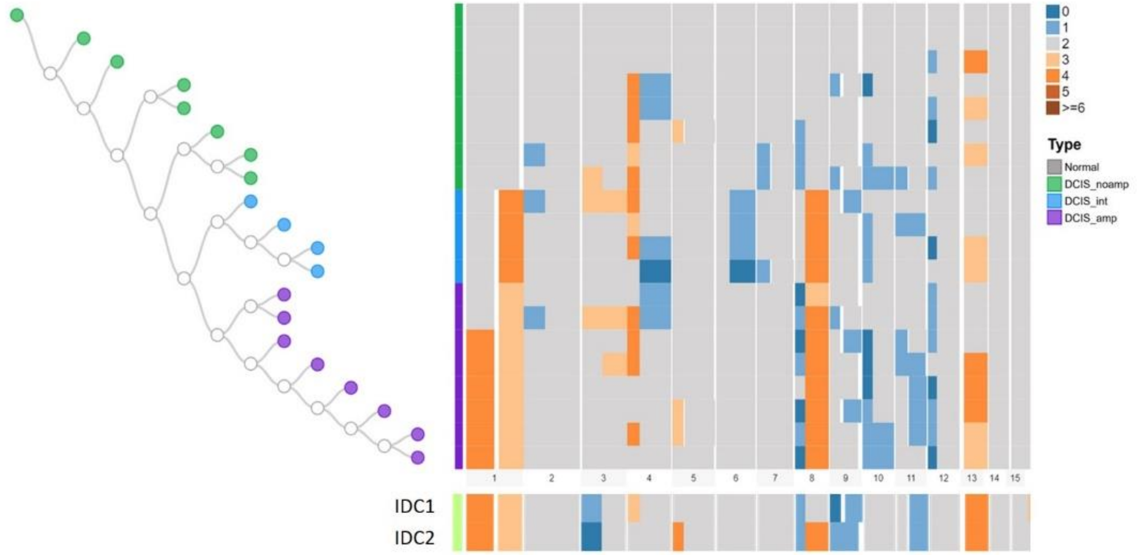

patient 10

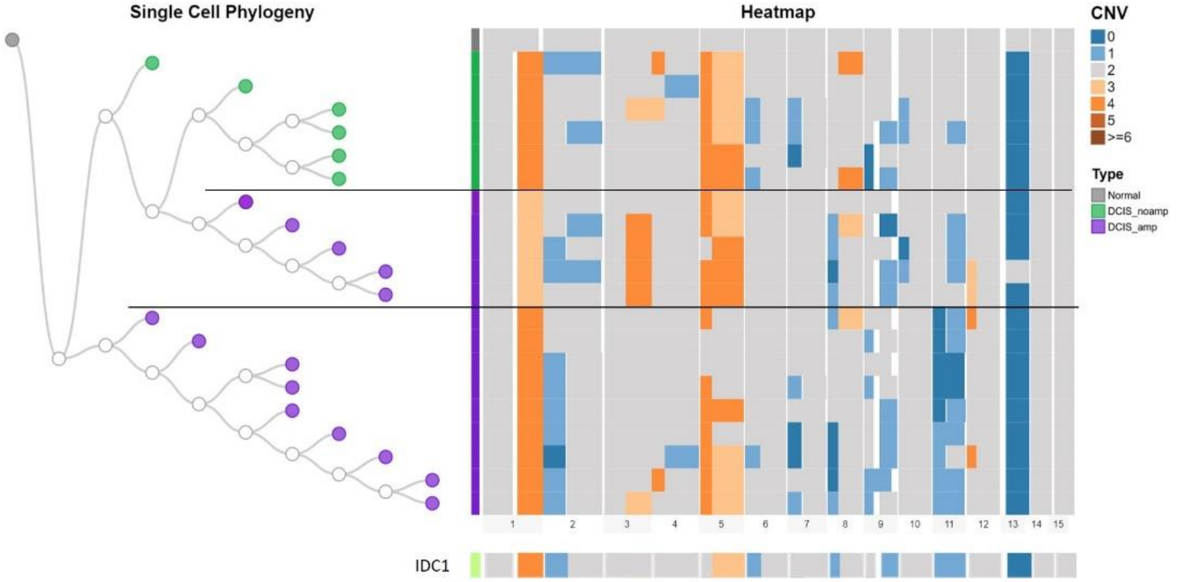

Supplementary Figure 13

Breast Invasive Carcinoma (TCGA, Cell 2015)

User-defined Patient List (**105** patients/samples) - ERBB2 amplification

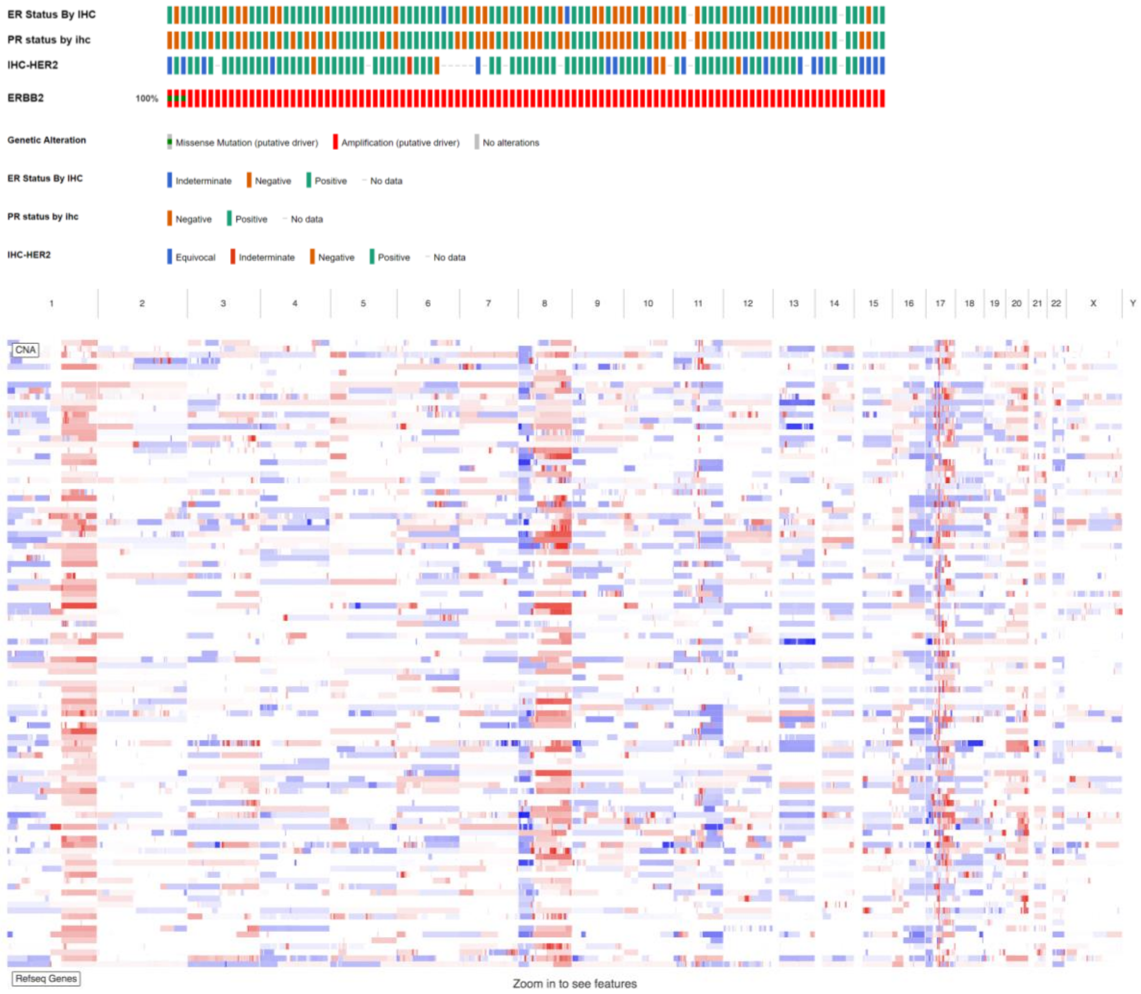

https://www.cancer.gov/**tcga**
https://www.cbioportal.org/

Supplementary Figure 14

Breast Invasive Carcinoma (TCGA, Cell 2015)

Whole-exome sequencing of 817 Breast Invasive Carcinoma tumor/normal pairs.

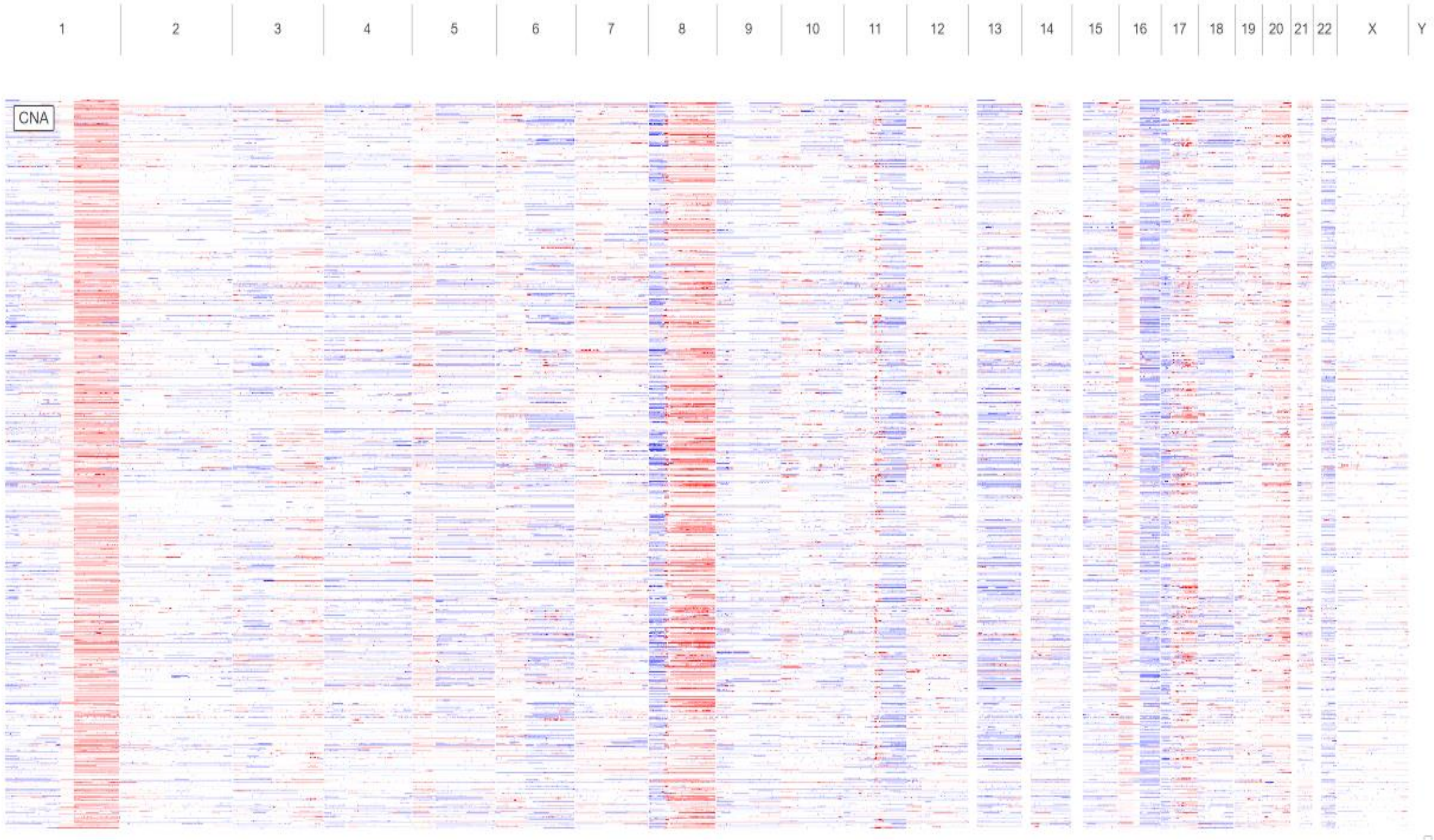

https://www.cancer.gov/**tcga**
https://www.cbioportal.org/

Supplementary Figure 15

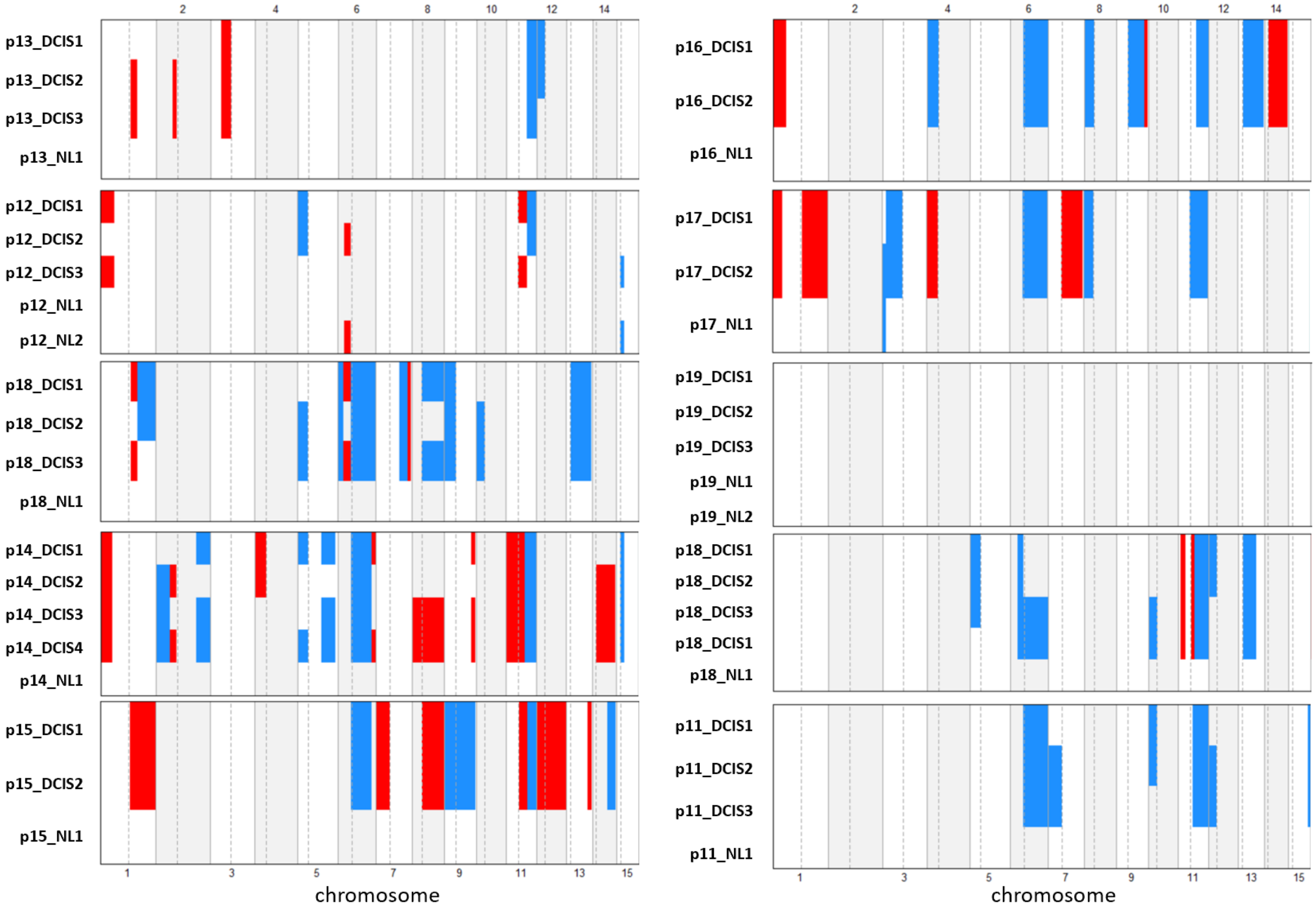

Supplementary Table 1. The posterior probability for a given cell with certain HER2-FISH signal/cell being non-amplified/amplified.

**HER2-FISH signal/cell**	**no_amp**	**amp**	**class**
0	0.55	0.41	no_amp
1	0.68	0.27	no_amp
2	0.67	0.28	no_amp
3	0.51	0.45	no_amp
4	0.30	0.65	amp
5	0.15	0.81	amp
6	0	0.95	amp
7	0	0.96	amp
8	0	0.96	amp
9	0	Inf	amp
10	0	Inf	amp

Supplementary Table 2. HER2-positive case summary

clinico-pathological information								samples			
**patient**	**ER**	**ER intensity**	**PR**	**PR intensity**	**age**	**grade**	**size (cm)**	**Normal**	**DCIS_noamp**	**DCIS_int**	**DCIS_amp**
p1	positive	100%, 3+	positive	30%, 2+	50	3	1.5	1	10	0	5
p2	negative	0%	negative	0%	55	3	1.1	1	2	0	6
p3	positive	80%, 3+	weak positive	1-10%, 1+	60	2	2.1	1	5	3	3
p5	positive	100%, 3+	positive	80%, 3+	56	2	0.9	1	7	4	8
p9	negative	0%	negative	0%	80	2	0.4	1	9	0	6
p10	positive	100%, 3+	negative	0%	48	3	4.2	1	5	0	14

Supplementary Table 4. Inflammatory score in DCIS_noamp and DCIS_amp

patient	Inflammation score	
	DCIS_noamp	DCIS_amp
p1	0	2
p2	1	1
p3	2	1
p5	1	1
p9	1	2
p10	0	1

Supplementary Table 5. HER2-negative case summary

clinico-pathological information								samples	
**patient**	**ER**	**ER intensity**	**PR**	**PR intensity**	**age**	**grade**	**size (cm)**	**Normal**	**DCIS_neg**
p11	positive	100%, 3+	positive	90%, 3+	34	2	4.5	1	3
p12	positive	100%, 3+	positive	100%, 3+	61	2	1.3	1	3
p13	positive	80%, 2+	positive	100%, 3+	48	1	2	1	3
p14	positive	100%, 3+	positive	90%, 2+	48	2	1.9	1	4
p15	positive	100%, 3+	positive	70%, 3+	58	2	3	1	2
p16	positive	100%, 3+	positive	90%, 2+	54	2	9.5	1	2
p17	positive	100%, 3+	positive	85%, 3+	66	2	3.6	1	2
p18	positive	100%, 3+	positive	30%, 2+	64	2	4.4	1	4
p19	positive	100%, 3+	positive	80%, 3+	41	2	7.2	1	4
p20	positive	100%, 3+	positive	100%, 3+	31	2	2.2	1	3
